# Intranasal Administration of GRP78 Protein (HSPA5) Confers Neuroprotection in a Lactacystin-Induced Rat Model of Parkinson’s Disease

**DOI:** 10.3390/ijms25073951

**Published:** 2024-04-02

**Authors:** Maria B. Pazi, Daria V. Belan, Elena Y. Komarova, Irina V. Ekimova

**Affiliations:** 1 Sechenov Institute of Evolutionary Physiology and Biochemistry, Russian Academy of Sciences, 44 Thorez pr., St. Petersburg 194223, Russia; daf205@yandex.ru; 2 Institute of Cytology, Russian Academy of Sciences, 4 Tikhoretsky pr., St. Petersburg 194064, Russia; elpouta@yahoo.com

**Keywords:** Parkinson’s disease, lactacystin, GRP78, ER stress, neuroprotection, substantia nigra, microglia

## Abstract

The accumulation of misfolded and aggregated α-synuclein can trigger endoplasmic reticulum (ER) stress and the unfolded protein response (UPR), leading to apoptotic cell death in patients with Parkinson’s disease (PD). As the major ER chaperone, glucose-regulated protein 78 (GRP78/BiP/HSPA5) plays a key role in UPR regulation. GRP78 overexpression can modulate the UPR, block apoptosis, and promote the survival of nigral dopamine neurons in a rat model of α-synuclein pathology. Here, we explore the therapeutic potential of intranasal exogenous GRP78 for preventing or slowing PD-like neurodegeneration in a lactacystin-induced rat model. We show that intranasally-administered GRP78 rapidly enters the substantia nigra pars compacta (SNpc) and other afflicted brain regions. It is then internalized by neurons and microglia, preventing the development of the neurodegenerative process in the nigrostriatal system. Lactacystin-induced disturbances, such as the abnormal accumulation of phosphorylated pS129-α-synuclein and activation of the pro-apoptotic GRP78/PERK/eIF2α/CHOP/caspase-3,9 signaling pathway of the UPR, are substantially reversed upon GRP78 administration. Moreover, exogenous GRP78 inhibits both microglia activation and the production of proinflammatory cytokines, tumor necrosis factor-α (TNF-α) and interleukin-6 (IL-6), via the nuclear factor kappa-light-chain-enhancer of activated B cells (NF-κB) signaling pathway in model animals. The neuroprotective and anti-inflammatory potential of exogenous GRP78 may inform the development of effective therapeutic agents for PD and other synucleinopathies.

## 1. Introduction

Parkinson’s disease (PD) is an age-related chronic neurodegenerative disorder ranking second in frequency after Alzheimer’s disease [1]. About 16 million people worldwide suffer from PD, and it is estimated that the number of PD patients will rise by 1.5–2-fold within the next 20–30 years due to the increase in centenarians [2]. The etiology of PD is largely unknown, with more than 90% of PD cases being sporadic [3]. Older age in combination with genetic profile and/or exposure to environmental pollution (herbicides, pesticides, infectious agents, etc.) are considered causative factors of sporadic PD onset and progression [4,5]. PD diagnosis relies on clinically significant symptoms, such as resting tremors, bradykinesia, muscular rigidity, and loss of balance. These symptoms are indicative of motor dysfunctions, which are associated with the degeneration of 50–60% of dopaminergic (DA) neurons in the substantia nigra pars compacta (SNpc), resulting in a reduction of dopamine in the striatum [6,7]. Such a delayed diagnosis of PD, when most specific neurons are already lost, explains the low effectiveness of existing PD therapies, primarily aimed at relieving symptoms. However, neuronal death also occurs in extranigral brain regions responsible for non-motor symptoms that may manifest in the pre-symptomatic (preclinical) PD stage, 20–30 years prior to the first motor symptoms [8]. A wide range of non-motor symptoms in PD includes sleep disorders, olfactory disturbances, enteral dysfunction, etc. [6]. Therefore, progress in treating PD is linked to the advancement of early diagnosis technologies and pathogenetically significant therapy, aiming to prevent or attenuate neurodegeneration in its early stage [9].

A pathological hallmark of PD is the overexpression and/or abnormal accumulation and misfolding of α-synuclein (α-syn) followed by the formation of Lewy bodies and Lewy neurites [10,11]. The Lewy bodies consist of up to 90% of α-syn phosphorylated at serine-129 (Ser129), and this post-translational modification appears to be associated with the formation and/or toxicity of aggregated proteins [12,13]. The intraneuronal accumulation of aberrant α-syn forms (misfolded and phosphorylated at Ser129) results from the dysfunction of both the ubiquitin–proteasome system (UPS) and the autophagy–lysosomal pathway [14,15,16]. The weakening of conformational control mechanisms ensured by heat shock proteins also contributes to protein aggregation [17,18,19]. The discovery of UPS functional insufficiency in familial and sporadic PD has promoted the development of novel in vivo PD models based on proteasome inhibitors, such as lactacystin (LC) [20,21]. LC is a metabolite of ubiquitous soil and water bacteria, e.g., Streptomyces lactacystinaeus. Given the lipophilic properties of LC and its ability to enter the human body with food, water, or dust and accumulate over time, exposure to this proteasome inhibitor can underlie some cases of PD [22]. When injected directly into the SNpc in rats, LC replicates the key neuropathological features of PD in the nigrostriatal and extranigral systems stage by stage, with the effects varying in a dose-dependent manner [21]. This sets LC apart from other neurotoxins used to model PD, making it one of the most promising substances for testing therapeutic strategies that can slow down neurodegeneration at various stages of PD development. In this study, we established an LC-induced model of early PD. This stage is particularly crucial for effective treatment since the majority of DA neurons are still preserved.

A growing body of evidence from animal models of PD and postmortem studies in PD patients suggests that the accumulation of α-syn oligomers can trigger neuronal death via the apoptosis pathway coupled with microglial activation, which contributes to the pathogenesis of progressive PD [10,23,24,25,26,27]. In addition to the cytosol of neurons, α-syn pathologically aggregates and accumulates in the endoplasmic reticulum (ER) lumen. This, in turn, induces ER stress, initiating an adaptive response through the activation of the unfolded protein response (UPR) [26,28,29,30,31,32]. The UPR includes three signaling pathways initiated by the PKR-like ER kinase (PERK), inositol-requiring transmembrane kinase/endoribonuclease 1α (IRE1α), or the activating transcription factor 6 (ATF6) [33]. A member of the 70 kDa heat-shock protein (HSP) chaperone family, the ER-associated 78 kDa glucose-regulated protein, also known as the immunoglobulin heavy chain-binding protein (GRP78/BiP), is a key regulator of the UPR signaling pathways. Under normal conditions, GRP78 binds IRE1α, PERK, and ATF6, maintaining their inactive state. When misfolded proteins accumulate within the ER, GRP78 binds them to prevent further misfolding and, thus, dissociates from the three ER stress receptors mediating the UPR. Biologically, the UPR aims to restore the ER function and protect cells against the toxic build-up of un-/misfolded proteins.

However, if ER stress is prolonged or exceeds the adaptive capacity of the cell, it can lead to the activation of apoptosis signaling and cell death. The PERK protein plays a crucial role in the regulation of ER-stress-induced apoptosis due to its involvement in many branching pathways [31,34]. Another important player is the C/EBP homologous protein (CHOP), a transcription factor and a downstream regulator of the PERK pathway. pPERK and its phosphorylated downstream target eukaryotic initiation factor 2α (eIF2a), both markers of UPR activation, are detected in neuromelanin-containing DA SNpc neurons in PD cases but not in age-matched controls [28]. The induction of the UPR has been documented in various in vitro and in vivo models of PD [26,30,35]. Overall, these findings indicate the involvement of the ER-stress PERK/CHOP pathway in neurodegeneration in PD. Therefore, modulating ER stress and inhibiting the PERK/CHOP-dependent pro-apoptotic UPR pathway could be a prospective therapeutic approach.

GRP78 chaperone is a specific modulator of ER stress, ensuring conformational control of nascent membrane-bound or secretory proteins. Unlike the cytosolic HSP members, GRP78 contains a signal sequence that targets it in the ER. In vivo and in vitro studies have shown that within cells containing α-syn aggregates, α-syn binds to the ER-stress sensor GRP78 [26,30]. This indicates that α-syn is a molecular target for GRP78. Chaperone GRP78 is a multifunctional protein that assists in a wide range of folding and refolding processes, the proteasomal endoplasmic-reticulum-associated protein degradation (ERAD) of misfolded proteins, maintaining calcium homeostasis, and regulating the UPR signaling [32,36]. Moreover, extracellular and exogenous GRP78 proteins demonstrate long-term anti-inflammatory and immunomodulatory properties in inflammatory diseases [37]. The literature data indicate that the mobilization of the GRP78-based chaperone mechanism serves as the first “line of defense” against the fatal consequences of α-syn toxicity and prolonged ER stress. The overexpression of GRP78 can reduce the death of DA neurons in the SNpc and loss of striatal dopamine by halting ER stress and apoptosis in an α-syn model of PD in rats [30,38].

These findings suggest that GRP78 can be a potential therapeutic target for the treatment of PD. Notably, GRP78 protein levels decrease in the SNpc with aging and in sporadic PD patients, which reflects the weakening of the protein conformational control and increased vulnerability of DA neurons to ER stress [38,39]. GRP78 is required for the survival of nigral neurons, and its lower level is suggested to be a predisposing factor for the onset and progression of PD and synucleinopathies in humans [38]. One of the ways to increase GRP78 in brain neurons is the intranasal delivery of the recombinant GRP78 protein. The intranasal method of GRP78 administration is informed by the in vitro data on the ability of exogenous GRP78 to penetrate living cells, translocate to the ER, and directly affect proteostasis and cell physiology [40]. Our preliminary experiments have shown that intranasal GRP78 administration helps mitigate the process of neurodegeneration in the nigrostrial system, suggesting the bioavailability of exogenous GRP78 [41]. This study aimed to develop a new neuroprotective approach to PD therapy through the intranasal administration of recombinant GRP78. We hypothesized that elevating GRP78 levels in the SNpc would prevent the abnormal accumulation and formation of pathological α-syn, modulate the UPR, block apoptosis, and inhibit microglial activation. Consequently, this would promote the survival of nigral neurons in the proteasome inhibitor-induced rat model of PD.

## 2. Results

### 2.1. GRP78 Treatment Prevents Neuronal Loss in the Substantia Nigra Pars Compacta in a Lactacystin-Induced Rat Model of Parkinson’s Disease

**Figure 1:**
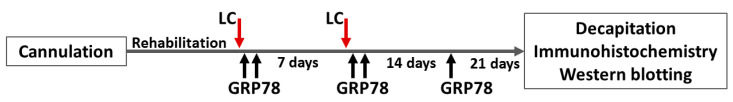
Experimental design. The timing and sequence of LC and GRP78 injections and procedures are shown. Red arrows, LC—microinjections of the proteasome inhibitor lactacystin into the SNpc (0.4 μg/1 μL). Black arrows, GRP78—treatment with recombinant human heat shock protein GRP78 (1.6 μg/8 μL) or the corresponding vehicle, sterile PBS, administered intranasally 4 h and 28 h following each microinjection of LC or vehicle and 7 days after the last injection.

To assess the therapeutic potential of exogenous GRP78, we used an LC-induced model of PD in rats, reproducing key pathological signs of PD [20,21]. At a dose of 0.4 μg, LC was injected into each side of the SNpc twice, with a 7-day interval (*n* = 8, group LC). Recombinant human GRP78 was administered intranasally (*n* = 8) at a dose of 1.6 μg/8 μL to each nostril 4 h and 28 h after each LC microinjection, as well as 7 days after the last microinjection (group LC+GRP78, see Figure 1 for details). Control rats (*n* = 8) were treated similarly but received an equivalent volume of the vehicle instead of LC and GRP78 (group vehicle). GRP78 was also introduced to naïve animals (*n* = 3, group GRP78). Twenty-one days after the first LC or vehicle microinjection, behavior tests were performed and then the animals were sacrificed for further immunohistochemical and biochemical analyses. In preliminary experiments [41], using motor behavior tests (sunflower seed test, Suok test, inverted horizontal grid test), we showed that double injections of 0.4 μg LC in the SNpc lead to no motor dysfunction. To assess the number of surviving DA neurons and their axons, brain sections were stained with antibodies against tyrosine hydroxylase (TH), the marker of DA neurons.

The morphological analysis of nigrostriatal sections revealed the loss of 27% of DA neurons in the SNpc and 19.4% of their axons in the dorsal striatum after LC administration compared to the vehicle (Figure S1). This suggests the development of neurodegeneration imitating the pre-symptomatic (preclinical) stage of PD, as motor dysfunction symptoms do not manifest until at least 50–60% of the DA neurons in the SNpc are lost [9]. Treatment with GRP78 significantly prevented the loss of DA neurons in the SNpc (Figure S1a,c) and their axons in the dorsal striatum (Figure S1b,d). On the other hand, GRP78 did not affect the number of TH-positive neurons in the SNpc in rats untreated with LC, indicating that GRP78 administration is not responsible for neurodegeneration. Thus, our data demonstrate that exogenous GRP78 mitigates the neurodegenerative process in the nigrostriatal system in the LC-induced rat model of PD, and chaperone therapy has a neuroprotective effect.

### 2.2. Exogenous GRP78 Can Penetrate Brain Structures and Be Internalized by Neurons and Microgliocytes in a Lactacystin Rat Model of Parkinson’s Disease

**Figure 2:**
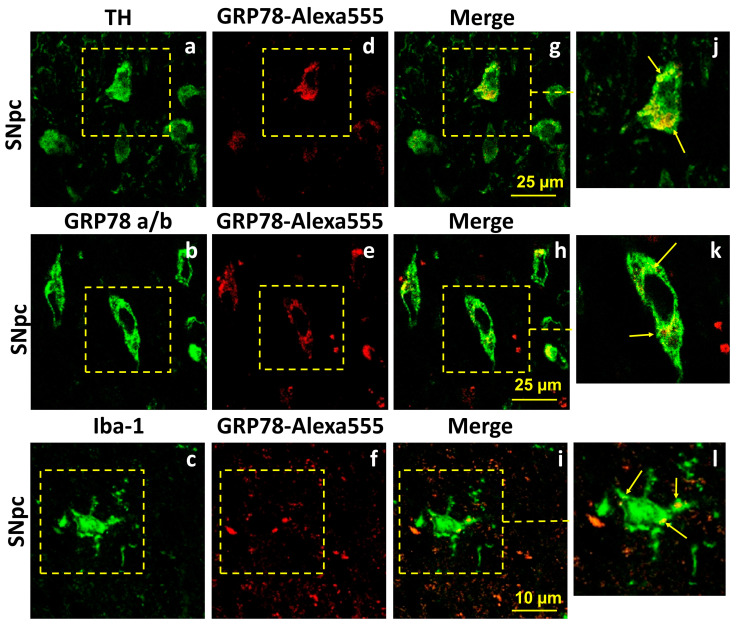
Labeled GRP78 penetrates the brain and is localized in DA neurons and microglial cells of the substantia nigra pars compacta (SNpc) 3 h after its intranasal administration in a rat model of Parkinson’s disease. GRP78 protein labeled by Alexa-555 was administered intranasally to rats (*n* = 4) after a microinjection of lactacystin as described in the Materials and Methods. Brain sections were stained with (**a**) specific anti-TH antibodies (green signal), (**b**) anti-Grp78 antibodies specific to human protein (green signal), and (**c**) anti-Iba-1 antibodies (green signal). (**d**–**f**) Localization of labeled GRP78 is seen as a red signal. (**g**–**i**) Panels show co-localization of labeled GRP78 and anti-TH, anti-Grp78, or anti-Iba-1 signals. (**j**–**l**) Panels show magnified representative images of the co-localization within neurons and microglia cells marked by yellow box. Arrows indicate co-localization of labeled GRP78 with corresponding proteins. Images were obtained using confocal microscopy. Scale bars are 25 μm for neurons in the SNpc and 10 μm for microglia.

The neuroprotective potential of exogenous GRP78 shown in the LC model of PD suggests its bioavailability to the brain upon intranasal administration. To experimentally prove that exogenous GRP78 can penetrate the brain and be internalized by cells when administered intranasally, we analyzed the localization of the fluorescently labeled protein in brain structures pathogenetically significant for PD. GRP78, labeled by Alexa-555, was administered intranasally to rats after the microinjection of LC and after the injection of the LC vehicle (phosphate buffer saline, PBS) into the SNpc. Using antibodies against TH, we found that labeled GRP78 (red signal, Figure 2d–j) penetrates the brain and localizes in the cytosol, but not in the nuclei of DA neurons of the SNpc 3 h after its intranasal administration, both after LC injections (Figure 2a,d,g,i) or after LC vehicle injection (Figure S2). The merged signal (yellow) illustrates the co-localization of labeled GRP78 and TH in cell bodies in the SNpc (Figure 2g,j). A red fluorescent signal was absent following the intranasal administration of unlabeled GRP78 to a rat receiving the LC microinjection into the SNpc (Figure S3).

Next, we stained SNpc brain sections with antibodies against GRP78 and evaluated the optical density of GRP78 in SNpc neurons in animals treated with Alexa-labeled GRP78 and control animals receiving its solvent (PBS). The analysis showed a clear trend towards increased protein levels GRP78 in neurons of the SNpc; the content of GRP78 increased 1.4 times (*p* = 0.06) 3 h after its administration compared with the control (Figure S4). This further confirms that GRP78 penetrates the brain and starts to accumulate in neurons 3 h after its intranasal administration.

Since proteins can undergo proteolysis in the brain, we stained the SNpc sections of rats that received Alexa-555-labeled GRP78 with antibodies against GRP78. We showed that GRP78, recognized by specific antibodies (green signal, Figure 2b), co-localized (the merged signal, yellow) with exogenous labeled GRP78 (Figure 2h,k). Importantly, exogenous GRP78 also migrated to other brain regions affected by PD in humans [8,42], such as the ventral tegmental area and locus coeruleus. There, GRP78 was able to cross the plasma membranes of neurons and localize in their cytosol, as illustrated in Figure S5. Thus, we can conclude that the loss of fewer DA neurons in the SNpc is linked to an increase in the content of the exogenous GRP78 protein after its intranasal administration and its neuroprotective properties in the LC-induced PD model.

As shown previously, exogenous GRP78 is rapidly internalized by monocytes in the peripheral blood, directly impacting various phenotypical and metabolic functions of myeloid cells [37,43]. We assumed that microglial brain cells could internalize exogenous GRP78, mediating its immunomodulatory effect. To test our assumption, we used antibodies against the microglial surface marker Iba-1 (ionized calcium-binding adaptor molecule) (Figure 2c). As seen in Figure 2f, GRP78 (red signal) was efficiently internalized by the microgliocytes of the SNpc (Figure 2i,l).

Thus, the protective effect of exogenous GRP78 on DA neurons of the SNpc appears to be associated with its ability to penetrate neurons and microglia and directly influence proteostasis and cell physiology during the development of PD-like pathology.

### 2.3. Exogenous GRP78 Prevents Abnormal Accumulation of Phosphorylated pS129-α-syn in Nigral Tissue in the Lactacystin Model of Parkinson’s Disease

In order to find out whether the protective effect of exogenous GRP78 on DA-ergic neurons is associated with a decrease in the signs of α-syn pathology, we analyzed the total content of the water-soluble α-syn protein and its phosphorylated form pS129 using a Western blot analysis with antibodies against α-syn and pS129-α-syn. We tested nigral tissue samples of rats, treated or untreated with GRP78, 21 days after the first LC microinjection (see the experimental scheme for details, Figure 1).

**Figure 3:**
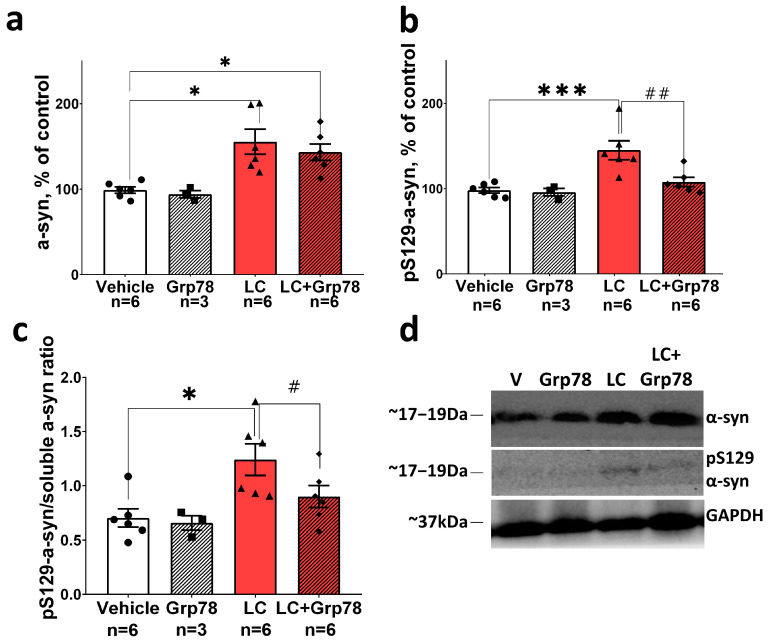
Exogenous GRP78 prevents abnormal accumulation of α-syn phosphorylated at S129 (pS129) in nigral tissue in rat model of Parkinson’s disease. Nigral content of (**a**) soluble form of α-syn, (**b**) α-syn phosphorylated at S129 (pS129), (**c**) phosphorylated to soluble α-syn ratio. Western blot analysis of nigral tissue was conducted with the antibodies against soluble and S129-phosphorylated forms of α-syn. Anti-GAPDH antibody staining was used as the loading control. (**d**) Representative Western blots are shown in panel. The results are presented as percentages of the control (100%) in (**a**–**c**) panels. Bar charts indicate mean values with standard errors. The dots, squares, triangles and rhombus show individual values per rat. Two-way ANOVA test followed by Tukey’s post hoc analysis were performed to determine the effects of GRP78 therapy. Asterisks indicate significant differences between groups according to Tukey’s post hoc tests: * *p* < 0.05, *** *p* < 0.001, vs. the vehicle group; # *p* < 0.05, ## *p* < 0.01, vs. the LC group. Interaction factor for soluble α-syn F (1, 15) = 7.329 *p* = 0.0162; Grp78 factor F (1, 15) = 14.34 *p* = 0.0018; LC (lactacystin) factor F (1, 15) = 13.63 *p* = 0.0022; Interaction factor for pS129-α-syn F (1, 17) = 4.982 *p* = 0.0394; Grp78 factor F (1, 17) = 6.399 *p* = 0.0216; LC factor F (1, 17) = 14.16 *p* = 0.0016.

Our results showed that the total concentration of water-soluble monomeric α-syn and its pS129 form in the SNpc of rats in the LC-induced PD model is 1.3 and 1.4 times higher, respectively, compared to the vehicle control (Figure 3a,b,d). As Figure 3c,d illustrate, the pS129/total soluble α-syn ratio increased with LC treatment. Therefore, pS129-α-syn predominates in the water-soluble monomeric α-syn fraction. At the same time point, the neurodegeneration of DA neurons in the SNpc and their axons in the dorsal striatum was observed after LC microinjections (Figure S1), which may be a consequence of the pS129-α-syn toxicity.

Treatment with GRP78 prevented the LC-induced accumulation of pS129 α-syn (Figure 3b–d), while levels of total water-soluble α-syn remained elevated (Figure 3a,d). This effect coincided with a better survival of TH-positive neurons in the nigrostriatal system (Figure S1). GRP78 did not change the amount of water-soluble α-syn protein and pS129 α-syn in LC-untreated control rats. Thus, our data demonstrate that the treatment of LC-animals with exogenous GRP78 can reduce the content of potentially cytotoxic pS129 α-syn form.

### 2.4. Exogenous GRP78 Counteracts the Activation of the GRP78/eIF2α/CHOP/Caspase-3,9 Pro-Apoptotic UPR Signaling Pathway in the Lactacystin Model of Parkinson’s Disease

To determine whether exogenous GRP78 counteracts the activation of the PERK/CHOP pro-apoptotic pathway of the UPR, we measured the GRP78 level and phosphorylation of eIF2α as ER stress indicators in nigral tissue using Western blot analyses. Furthermore, we assessed the levels of a pro-apoptotic transcription factor CHOP and well-known effectors of neuronal apoptosis—cleaved forms of caspase-3 and caspase-9—which play a crucial role in cell degeneration through the canonical mitochondrial apoptosis pathway.

**Figure 4:**
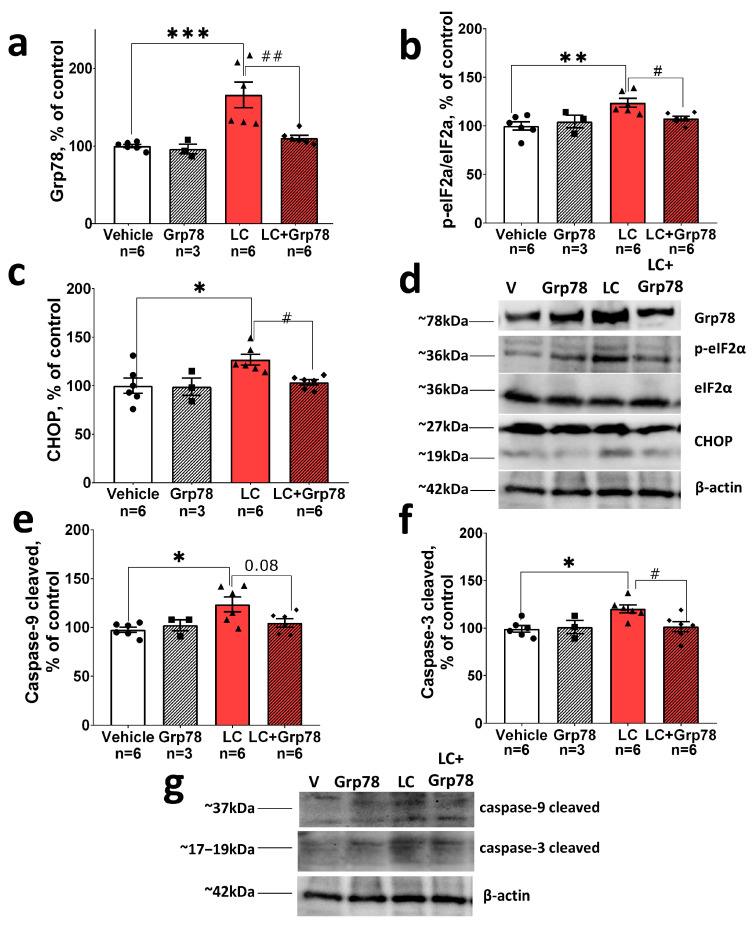
Exogenous GRP78 blocks the pro-apoptotic GRP78/eIF2α/CHOP/caspase-3,9 signaling pathway of the UPR in nigral tissue in a rat model of Parkinson’s disease. Nigral content of (**a**) GRP78, (**b**) phosphorylated to total eIF2α, (**c**) CHOP. (**d**) Representative immunoblots. Nigral content of (**e**) cleaved caspase-9, (**f**) cleaved caspase-3. (**g**) Representative immunoblots. Western blot analysis of the nigral tissue was conducted with the antibodies against GRP78 (1:1000, rabbit, Abcam, Cambridge, UK), eIF2a (1:750, rabbit, Affinity Biosciences, Zhenjiang, China), pSer51-eIF2a (1:1000, rabbit, Abcam, Cambridge, UK), CHOP (1:1000, rabbit, Affinity Biosciences, Zhenjiang, China), cleaved caspase-9 (1:1000, rabbit, Affinity Biosciences, Zhenjiang, China), and cleaved caspase-3 (1:1000, rabbit, Affinity Biosciences, Zhenjiang, China). Staining with anti-β-Actin antibodies (1:1000, mouse, Santa Cruz Biotechnology, Dallas, TX, USA) was used as the loading control. The results are presented as percentages of the control (panels (**a**–**c**,**e**,**f**)). Bar charts indicate mean values with standard errors. The dots, squares, triangles and rhombus indicate individual values per rat. Two-way ANOVA test followed by Tukey’s post hoc analysis were performed to determine the effects of GRP78 therapy. Asterisks indicate significant differences between groups according to Tukey’s post hoc tests: * *p* < 0.05; ** *p* < 0.01. *** *p* < 0.001, vs. the vehicle group; # *p* < 0.05, ## *p* < 0.01, vs. the LC group. Interaction factor for GRP78 F (1, 20) = 8.83 *p* = 0.0076; Grp78 treatment factor F (1, 20) = 7.413 *p* = 0.0131; LC (lactacystin) factor F (1, 20) = 15.89 *p* = 0.0007. Interaction factor for p-eIF2a/eIF2a ratio F (1, 19) = 5.68 *p* = 0.0278; Grp78 treatment factor F (1, 19) = 1.874 *p* = 0.1869; LC factor F (1, 19) = 7.819 *p* = 0.0115. Interaction factor for CHOP F (1, 16) = 3.126 *p* = 0.0961; Grp78 treatment factor F (1, 16) = 3.672 *p* = 0.0734; LC factor F (1, 16) = 47.898 *p* = 0.0418. Interaction factor for cleaved caspase-9 F (1, 17) = 4.124 *p* = 0.0582; Grp78 treatment factor F (1, 17) = 1.513 *p* = 0.2355; LC factor F (1, 17) = 5.911 *p* = 0.0264. Interaction factor for cleaved caspase-3 F (1, 17) = 6.417 *p* = 0.0214; Grp78 treatment factor F (1, 17) = 5.646 *p* = 0.0295; LC factor F (1, 17) = 7.451 *p* = 0.0143.

Using specific antibodies against GRP78 and against total and phosphorylated (Ser51) forms of eIF2α, we found that the GRP78 protein level increased by 66 ± 13.2% (*p* ≤ 0.001) in the SNpc on day 21 after the first LC injection compared to the vehicle control (Figure 4a,d). eIF2a phosphorylation (Ser51) also increased in the LC-treated animals, which suggests that there is ER stress in the SNpc (Figure 4b,d). Next, we investigated whether the upregulation of pSer51-eIF2 coincided with elevated levels of pro-apoptotic factors in the SNpc, such as CHOP and cleaved caspase-9 and caspase-3. We found that the CHOP protein was upregulated in LC-injected animals compared to the vehicle control (Figure 4c,d). We also observed a 23 ± 7% increase in cleaved caspase-9 protein levels (*p* ≤ 0.05) and a 24 ± 4.8% increase in cleaved caspase-3 levels (*p* ≤ 0.01) after LC administration (Figure 4e–g), indicating the activation of the pro-apoptotic PERK-CHOP branch of the UPR and the development of neuronal apoptosis induced by ER stress.

In contrast, treatment with GRP78 downregulated ER stress mediators and the level of pro-apoptotic proteins in LC-injected animals. The Western blot assessment showed no increase in the levels of GPR78 or pSer51-eIF2a in the SNpc (Figure 4b,d). Moreover, GRP78 prevented the upregulation of the pro-apoptotic factor CHOP, as evidenced by a decrease in CHOP protein levels compared to control values (Figure 4c,d). We also found that the levels of cleaved caspase-9 and cleaved caspase-3 returned to normal in the SNpc after GRP78 administration (Figure 4e–g). There was no change in the GRP78 content in the group of GRP78-treated animals. This is due to the fact that the content of GRP78 was measured 7 days after the last administration of exogenous GRP78. Thus, we suggest that by this time point, the administered exogenous GRP78 protein degraded. Consequently, in our experiments in the LC+GRP78 group, the elevation of GRP78 content was not observed for two reasons: (i) exogenous GRP78 had degraded by this time; (ii) treatment with GRP78 prevented the development of ER stress; therefore, the expression of endogenous GRP78 did not occur.

At the same time, the Western blot analysis demonstrated no significant changes in GRP78, p-eIF2, CHOP, activated caspase-3, and caspase-9 in the SNpc in control (LC-untreated) rats. This indicates that GPR78 itself does not induce ER stress or apoptosis in healthy animals.

In summary, our results showed that the intranasal administration of GRP78 prevented the activation of the GRP78/eIF2/CHOP signaling pathway, caspase-9, and caspase-3. This inhibition effectively mitigated the ER stress response and reduced apoptosis in the SNpc in the LC model of the preclinical stage of PD in rats.

### 2.5. Exogenous GRP78 Inhibits Microglia Activation and the Production of Proinflammatory Cytokines TNF-α and IL-6 via the NF-κB Signaling Pathway in the Lactacystin Model of Parkinson’s Disease

**Figure 5:**
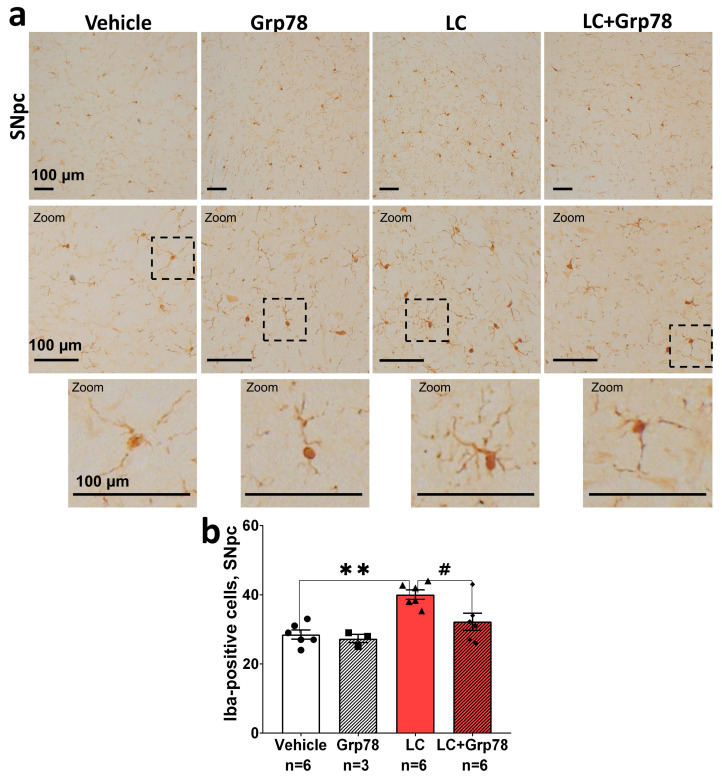
Exogenous GRP78 inhibits microglia activation in a lactacystin rat model of Parkinson’s disease. (**a**) Brain sections (10 µm) of the substantia nigra pars compacta (SNpc, (**a**)) were prepared according to the brain atlas and stained with antibodies against Iba-1 (1:500; rabbit, Novus Biologicals, Centennial, CO, USA). The images were obtained using a Zeiss Axio Imager A1 microscope (Carl Zeiss, Oberkochen, Germany) with a built-in video camera and Axio-Vision 4.8 software. Original images are shown in the upper panel. Scale bars are 100 μm. The second panel show magnified images of microglia cells (zoom). The third panel show magnified images of microglia morphology of cells within dotted box area (zoom). (**b**) Quantitative analysis was performed using 10–12 sections from each animal at the same level of the studied zones, separated by approximately 70 μm. The number of cells accounted for a standard area of tissue captured by a light microscope camera using ×20 lens. The analysis was performed using the PhotoM freeware version 1.21 (http://www.t_lambda.chat.ru/ accessed on 11 December 2019). Bar charts indicate mean values with standard errors. The dots, squares, triangles and rhombus show individual values per rat. Two-way ANOVA test followed by Tukey’s post hoc analysis were performed to determine the effects of GRP78 therapy. Asterisks indicate significant differences between groups according to Tukey’s post hoc tests: ** *p* < 0.01 vs. the vehicle group; # *p* < 0.05 vs. the LC group. Interaction factor for microglia in SNpc F (2, 23) = 2.099; Grp78 factor F (2, 23) = 5.466 *p* = 0.0284; LC factor F (2, 23) = 17.04 *p* = 0.0004.

We then investigated whether exogenous GRP78 has anti-inflammatory properties. As an increased number of activated microgliocytes is a marker of neuroinflammation [44], we first assessed the status of microglia in the SNpc of LC-treated rats. For this purpose, we implemented immunohistochemistry using antibodies against the microglial marker Iba-1 to quantify the number of Iba-1-immunopositive cells. We showed that LC caused a 38% (*p* = 0.002) increase in Iba-1-positive cells in the SNpc on day 21 after the first injection compared to the vehicle control (Figure 5a,b). During the visual analysis under a light microscope, we observed LC-induced morphological changes, such as larger soma sizes and less ramified processes (Figure 5a, lower panel). This indicates an increase in the number of microglial cells adopting an activated phenotype.

**Figure 6:**
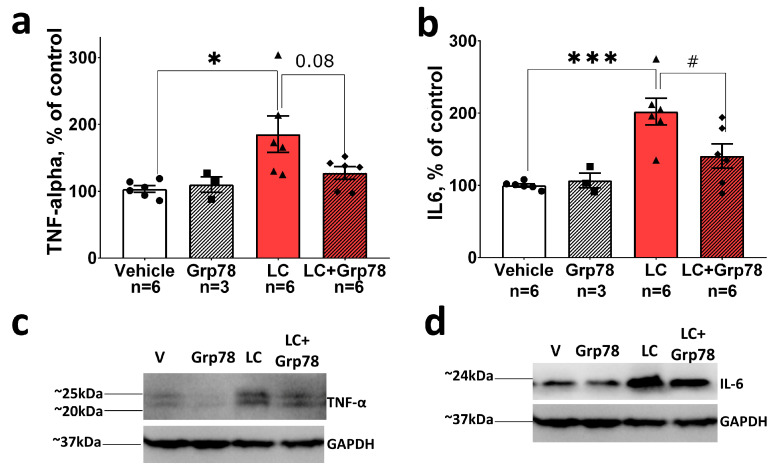
Exogenous GRP78 inhibits the production of pro-inflammatory cytokines TNF-α and IL-6 in a lactacystin rat model of Parkinson’s disease. Content of (**a**) TNF-α and (**b**) IL-6 in SNpc tissue in the LC-induced model of PD. (**c**,**d**) Representative Western blots. Western blot analysis was conducted with the antibodies against TNF-α (1:1000, rabbit, Affinity Biosciences, Zhenjiang, China), IL-6 (1:1000, rabbit, Affinity Biosciences, Zhenjiang, China). Anti-GAPDH antibodies (1:1000, mouse, Affinity Biosciences, Zhenjiang, China) were used for loading control. Bar charts indicate mean values with standard errors. The dots, squares, triangles and rhombus show individual values per rat. Two-way ANOVA test followed by Tukey’s post hoc analysis were performed to determine the effects of GRP78 therapy. Asterisks indicate significant differences between groups according to Tukey’s post hoc tests: * *p* < 0.05, *** *p* < 0.001, vs. the vehicle group; # *p* < 0.05, vs. the LC group. Interaction factor for TNF-α F (1, 17) = 3.228 *p* = 0.0902; Grp78 treatment factor F (1, 17) = 2.034 *p* = 0.1719; LC (lactacystin) factor F (1, 17) = 7.614 *p* = 0.0134. Interaction factor for IL-6 F (1, 17) = 4.896 *p* = 0.0409; Grp78 treatment factor F (1, 17) = 3.164 *p* = 0.0932; F (1, 17) = 19.58 *p* = 0.0004.

Next, we investigated whether the activation of microglia is associated with the release of pro-inflammatory cytokines TNF-α and IL-6, which participate in the pathogenesis of PD [44]. The immunoblot analysis demonstrated that the levels of TNF-α and IL-6 in the SNpc increased by ~2 times in LC-injected rats compared to the control (Figure 6). Taken together, these data indicate the development of the inflammatory process in the SNpc coupled with the death of DA neurons in the LC-induced rat model of the preclinical stage of PD.

In contrast, treatment with GRP78 decreased reactive microgliosis, as indicated by a 20% (*p* < 0.05) decrease in Iba-1-positive cells (Figure 5), and inhibited the production of pro-inflammatory cytokines TNF-α and IL-6 (Figure 6) in the SNpc of model animals. The administration of GRP78 alone affected neither the number of Iba-1-positive cells nor TNF-α and IL-6 levels in the SNpc. The results show that GRP78 can provide neuronal protection against the excessive activation of microglia in the LC-induced rat model of PD.

**Figure 7:**
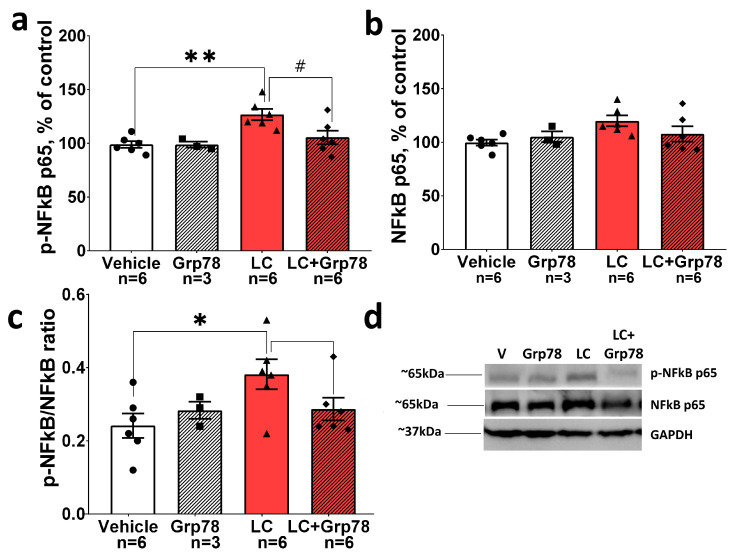
GRP78 can protect neurons from the excessive activation of microglia via NF-κB signaling pathways in the LC-induced rat model of PD. Content of (**a**) NF-κB p65, (**b**) phosphorylated NF-κB p65 (Ser536), and (**c**) phosphorylated to total NF-κB p65 ratio in SNpc in LC model of PD. (**d**) Representative immunoblots. Western blot analysis of the nigral tissue was conducted with the antibodies against NF-κB p65 (1:1000, mouse, Cell Signaling, Danvers, MA, USA), and phosphorylated NF-κB p65 (Ser536) (1:1000, rabbit, Cell Signaling, Danvers, MA, USA). Anti-GAPDH antibodies (1:1000, mouse, Affinity Biosciences, China) were used for loading control. Bar charts indicate mean values with standard errors. The dots, squares, triangles and rhombus show individual values per rat. Two-way ANOVA test followed by Tukey’s post hoc analysis were performed to determine the effects of GRP78 therapy. Asterisks indicate significant differences between groups according to Tukey’s post hoc tests: * *p* < 0.05, ** *p* < 0.01, vs. the vehicle group; # *p* < 0.05 vs. the LC group. Interaction factor for total NF-κB F (1, 17) = 2.337 *p* = 0.1447; Grp78 treatment factor F (1, 17) = 0.3563 *p* = 0.5584; LC factor F (1, 17) = 3.978 *p* = 0.0624. Interaction factor for phosphorylated NF-κB (Ser536) F (1, 17) = 3.776 *p* = 0.0687; Grp78 treatment factor F (1, 17) = 3.897 *p* = 0.0648; LC factor F (1, 17) = 10.29 *p* = 0.0052. Interaction factor for p-NF-κB (Ser536)/NF-κB ratio F (1, 17) = 5.358 *p* = 0.0334; Grp78 treatment factor F (1, 17) = 1.339 *p* = 0.2631; LC factor F (1, 17) = 2.477 *p* = 0.1339.

To establish the mechanism enabling the GRP78-mediated inhibition of the microglia activation, we explored the activity of the NF-κB-dependent p65/RelA signaling pathway. This pathway facilitates the induction of proinflammatory cytokines [45] and NF-κB dysregulation has been found in patients with PD and in the substantia nigra of MPTP-treated mice [46]. Post-mortem studies showed an increase p65 nuclear translocation in melanized neurons of the substantia nigra that is supportive of NF-κB activation in PD. We assessed the expression patterns of p65 and phosphorylated-p65 (p-p65) after LC treatment with or without GRP78 using Western blot analysis. The level of p-p65 was found to increase in the LC group only, suggesting the activation of NF-κB during PD development. However, GPR78 inhibited the increase of p-p65 expression in the SNpc in the LC model (Figure 7), while no significant changes were found in LC-untreated rats. Hence, the decrease in p65 phosphorylation can be an essential factor in inhibiting activated microglia by exogenous GRP78.

Taken together, our data demonstrate that GRP78 can protect neurons from the excessive activation of microglia via NF-κB signaling pathways in the LC-induced rat model of PD.

## 3. Discussion

With the population aging rapidly, the global prevalence of PD is rising, which significantly contributes to the increase in healthcare costs. Developing preventive PD therapy has proven challenging due to the limited bioavailability of neuroprotective drugs, partly because of the blood–brain barrier. One of the new approaches is the intranasal route of administration, delivering the drug from the nasal cavity directly to the brain via the olfactory and trigeminal nerves. It allows neurotherapeutic agents, including both small and large molecules, to bypass the blood–brain barrier [47,48]. Intranasal administration has shown therapeutic effects in animal and human studies of different pathologies [48].

In this study, we evaluated the neuroprotective potential of the intranasally administered recombinant human protein GRP78 in a rat PD model. The intranasal route was chosen considering that GRP78 protein can leave the ER, traverse the cell membrane, and enter the extracellular space [49], cerebrospinal fluid, and peripheral blood under normal and pathological conditions [37,50,51,52]. Moreover, when administered intravenously, exogenous GRP78 or its synthetic analog IRL201805 can be rapidly internalized by monocytes in the peripheral blood and directly impact various phenotypical and metabolic functions of myeloid cells [37]. It is noteworthy that the ability to enter the mammalian brain and neurons has been observed for another member of the same chaperone family, HSP70 (HSPA1). After intranasal administration, human recombinant HSP70 demonstrates therapeutic effects in animal models of PD and Alzheimer’s disease [53,54]. This highlights the potential of using the intranasal delivery of chaperones to the brain for neuroprotection.

Neuroprotective interventions are most effective at the early (preclinical) stage of the pathological process. Therefore, we utilized a previously developed LC-induced model [21,55] that reproduces the main pathogenetic signs of the preclinical stage of PD in rats. These include the degeneration of 27% of DA neurons in the SNpc (a level that is characteristic of the preclinical PD stage [8] (Figure S1), development of α-syn pathology (Figure 3), and signs of chronic neuroinflammation (Figure 5 and Figure 6)). At the molecular level, the model is characterized by the activation of the pro-apoptotic GRP78/PERK/eIF2/CHOP UPR pathway, caspases-9 and -3 (Figure 4), and the NF-κB-dependent p65 inflammatory signaling pathway (Figure 7). Yet, no motor dysfunction is detected.

At the first stage of our research, we demonstrated that intranasally administered GRP78 penetrates the mammalian brain and is internalized by DA neurons in the SNpc and other brain regions that can be affected by PD in humans (Figure S2). In addition, we have shown that exogenous GRP78 penetrates the brain under normal conditions (Figure S2) and accumulates in the neurons of the SNpc 3 h after administration (Figure S4), but 7 days after administration, GRP78 degrades, since its concentration in the SNpc tissue does not change in comparison to control animals (Figure S4). The internalization of GRP78 is assumed to occur through nonspecific or receptor-mediated endocytosis [37]. However, it is unclear what specific receptors and/or docking proteins facilitate endocytosis.

Next, we showed that GRP78 treatment mitigated the process of neurodegeneration in the rat model that mimics the preclinical stage of PD. It is evidenced by an increase in the number of TH-positive neurons in the SNpc and TH-positive axons in the dorsal striatum (Figure S1). Furthermore, intranasal treatment with GRP78 in control animals, without LC, was characterized by neither neurodegeneration in the nigrostriatal system nor behavioral deficit. This indicates the absence of cytotoxic properties of GRP78. Similar neuroprotective effects of elevating GRP78 via its overexpression in the SNpc have been shown in α-syn pathology models in rats [30,38], the 1-methyl-4-phenyl-1,2,3,6-tetrahydropyridine (MPTP) PD model in mice [56], and the rotenone PD model in rats [57]. Taken together, our data, along with the existing literature, indicate the therapeutic significance of elevating GRP78 levels in the brain during the development of PD-like pathology.

The abnormal accumulation of α-syn, and especially its phosphorylated and oligomeric forms, in the ER lumen and cytosol of the SNpc DA neurons is known to play a critical role in neuronal death in PD, although the underlying mechanism is poorly understood [10,58,59]. We first focused on investigating whether the neuroprotective effect of exogenous GRP78 is related to its ability to prevent α-syn pathology and the induction of the pro-apoptotic ER stress branch. We assessed levels of total and phosphorylated α-syn accumulated in the SNpc in the LC-induced PD model (Figure 3a,b) and demonstrated that pS129-α-syn predominated in the water-soluble monomeric α-syn fraction (Figure 3c). Hence, the enhanced S129-phosphorylation of α-syn may play a key role in the death of DA neurons in the SNpc. This assumption is supported by evidence showing that blocking S129-phosphorylation results in fewer α-syn aggregates and reduces neuronal cell death induced by the mitochondrial toxin rotenone [59]. In addition, the increased phosphorylation of α-syn can contribute to its transformation into oligomers or even aggregates, thereby affecting the cytotoxicity of α-syn and promoting neuronal death [12,59,60,61]. It is assumed that extensive S129 phosphorylation during PD-like pathology is most likely caused by an increased influx of extracellular Ca^2+^ due to mitochondrial impairment [62] and the increased expression and activity of polo-like kinase 2 (PLK2, also known as serum-inducible kinase or SNK) [63,64].

Here, we showed that treatment with GRP78 reduced the content of the pS129 α-syn form (Figure 3b–d) in the LC model of PD. This effect coincided with an increase in the survival of DA neurons in the SNpc. We suggest that, at least in part, it was mediated by the direct interaction of GRP78 with both phosphorylated and non-phosphorylated forms of α-syn [26,29,30]. Such interaction could prevent excessive S129-phosphorylation and inhibit the multistep aggregation pathway of α-syn, reducing related toxicity.

The accumulation of aberrant α-syn forms is a central element for the induction of the UPR that can trigger apoptotic cell death in PD [31]. Since the exogenous GRP78 protein prevented the development of α-syn pathology in our preclinical PD model in rats, we then tested the hypothesis that this effect can lead to the inhibition of the ER stress response and a reduction of apoptosis in the SNpc. Indeed, the accumulation of monomeric α-syn in the SNpc correlated with the activation of the pro-apoptotic PERK-dependent pathway of the UPR in LC-treated rats (Figure 4a–d). This was evidenced by an increase in the level of the sensor protein and UPR activator GRP78, as well as the activation of eIf2α and upregulation of the ATF4-dependent pro-apoptotic factor CHOP (Figure 4a–d). At the same time, CHOP upregulation resulted in the activation of caspase-9 and caspase-3 (Figure 4e–g), promoting cell degeneration through the canonical mitochondrial apoptosis pathway. Overall, our data indicate that the prolonged hyperactivation of the PERK/CHOP pathway of the UPR promotes ER stress-dependent apoptosis in the SNpc in the animal model of preclinical PD. These findings correlate with studies on postmortem tissues from PD patients [28,33] and animal models of PD [35,65,66,67] that demonstrate the activation of the pro-apoptotic PERK/CHOP pathway in nigral tissue. Treatment with GRP78 downregulated ER stress mediators of the PERK-dependent pathway of the UPR (Figure 4a–d) and prevented the activation of pro-apoptotic caspases-9 and -3 (Figure 4e–g), which contributed to the survival of DA neurons in the SNpc in LC-injected animals. These results support the data on the neuroprotective effect of GRP78 overexpression, which is associated with the downregulation of the pro-apoptotic factor CHOP and a reduction in apoptosis in the SNpc in the rat model of α-syn pathology [30].

Neuroinflammation manifests in microglia activation and lymphocyte infiltration. It can be provoked by the release of misfolded α-syn from damaged and dead neurons, leading to the development and progression of PD [23]. Activated microglia is a chronic source of pro-inflammatory cytokines, reactive oxygen species (ROS), and nitric oxide (NO), all of which can induce neuronal death [68]. Large numbers of activated microglia and elevated levels of TNF-alpha receptor R1 in the SNpc, along with activated caspase-1 and caspase-3, have been observed in PD [69,70,71]. Furthermore, in vivo imaging has confirmed that widespread microglial activation is associated with the pathological process in idiopathic PD [45]. In our LC-induced model of the preclinical PD, the number of Iba-1 positive cells of amoeboid-like phenotype (Figure 5) was correlated with an increase in pro-inflammatory cytokines TNF-α and IL-6 (Figure 6) in the SNpc. This indicates the development of reactive microgliosis and neuroinflammation, potentially contributing to the death of DA neurons. We showed that intranasally delivered GRP78 was efficiently internalized by the microgliocytes of the SNpc (Figure 2c,f,i,l) and directly affected cell physiology. Its protective action manifested in a decreased number of Iba1-positive cells and lower levels of TNF-α and IL-6; these data indicate reduced microglial activation and neuroinflammation in the SNpc. However, it is not yet clear whether the anti-inflammatory effect of GRP78 is associated with the phenotypic shift of pro-inflammatory M1 microglia to anti-inflammatory M2 microglia, which may promote neuroprotection. Notably, following the systemic administration of GRP78 or its analog IRL201805 in an animal model of rheumatoid arthritis, these proteins are rapidly internalized by monocytes, which eventually leads to an increased secretion of IL-10 and the suppression of TNF-α and IL-1β release [37]. These anti-inflammatory properties of exogenous GRP78 help regulate and resolve chronic inflammation.

Toll-like receptors (TLRs) can serve as essential immune receptors in PD, triggering neuroinflammation [72,73]. TLRs can recognize a wide variety of damage-associated molecular patterns, including misfolded α-syn, released by damaged and dead neurons. Upon the recognition of these molecules, TLRs trigger a signaling cascade that activates NF-κB factors. NF-κB factors play a crucial role in the regulation of inflammation and apoptosis, and are involved in the pathogenesis of PD [46]. To find out whether the anti-inflammatory effect of exogenous GRP78 depends on its ability to modulate the NF-κB signaling pathway, we assessed expression patterns of p65 and phosphorylated-p65. The NF-κB-dependent p65 signaling pathway was shown to be activated in the SNpc in the LC-induced model of preclinical PD. This may be a signal regulating the molecular activation of microglia at an early stage of the disease. However, treatment with GRP78 inhibited the nigral activation of NF-κB (Figure 6). Taken together, the results demonstrate that exogenous GRP78 exerts potent anti-inflammatory effects. It can protect neurons against the excessive activation of microglia by targeting NF-κB signaling pathways during the development of LC-induced PD-like pathology.

Overall, our data support the therapeutic relevance of delivering GRP78 intranasally to the brain to prevent and/or slow down PD-like neurodegeneration. We determined that the neuroprotective potential of exogenous GRP78 is linked to its ability to (i) prevent the manifestation of α-syn pathology, (ii) block ER stress-dependent apoptosis, and (iii) mitigate the excessive activation of microglia by targeting NF-κB signaling pathways in the LC-induced rat model of preclinical PD.

## 4. Materials and Methods

### 4.1. Animals

The study was carried out in 6-month-old male Wistar rats, weighing 280–310 g. The animals were housed in individual cages under standard environmental conditions (12:12 h light–dark cycle; ambient temperature 23 ± 2 °C; food and water available ad libitum). The experiments were conducted under the requirements of the EU Directive 2010/63/EU on the treatment of laboratory animals and those of the Sechenov Institute of Evolutionary Physiology and Biochemistry of the Russian Academy of Sciences (Protocol No # 1-17/2022, 27 January 2022). The rats were placed in groups randomly.

### 4.2. Implantation of Guiding Cannulas

Before implantation surgery, animals were anesthetized with intramuscular injection of Zoletil-100 (50 mg/kg; tiletamine hydrochloride and zolazepam; Virbac, Carros, France) and then placed into a stereotaxic device (Narishige, Tokyo, Japan). Two stainless-steel guide cannulas (internal diameter 0.3 mm) were implanted into the SNpc for bilateral drug injections. The coordinates were as follows: 5.0 mm caudal to the bregma, 2.0 mm lateral to the midline, and 7.5 mm deep from the skull surface [74]. The guide cannulas were secured with Akrodent dental cement (Stoma, Kharkiv, Ukraine). Then, animals were returned to their home cages, and experiments began no earlier than 7 days post-surgery.

### 4.3. Modeling Parkinson’s Disease in Wistar Rats

Cannulated animals were used to model PD and evaluate the protective potential of intranasally administered GRP78. To create the PD model, we used a specific, irreversible proteasome inhibitor lactacystin (LC; Enzo Life Sciences, Farmingdale, NY, USA). Phosphate-buffered saline (PBS) was filtered through a sterilized syringe filter (30 mm, PVDF, 0.22 μm; JET BIOFIL^®^, Seoul, Republic of Korea). LC was diluted in sterile PBS to the final concentration of 0.4 μg/μL and injected immediately after dilution through cannulas to rats (*n* = 6–8, group LC). Two sequential bilateral microinjections of LC into the SNpc were performed at a dose of 0.4 μg/1 µL with a weekly interval. For microinjections, we used a needle with an external diameter of 0.2 mm attached to a 1 μL Hamilton syringe (Hamilton, Reno, NV, USA) via a short length of polyethylene tubing. LC was injected at a flow rate of 0.1 μL/ min. Control rats were treated similarly but received an equivalent volume of vehicle (PBS) instead of LC.

### 4.4. GRP78 Treatment

Recombinant human heat shock protein GRP78 (Sigma, Livonia, MI, USA) was diluted in sterile PBS (pH 7.4) and administered intranasally (to each nostril) to rats (*n* = 6–8, group LC+GRP78) at a dose of 1.6 μg/8 μL, 4 h and 28 h after each microinjection of LC. Additional administration of GRP78 was performed 7 days after the last LC microinjection. The control group of animals (*n* = 6–8) received an equivalent volume of the vehicle (PBS) instead of LC and GRP78. GRP78 was also administered to intact animals, untreated with LC (*n* = 3, group GRP78) (see experimental design in Figure 1). Intranasal injections were performed using a 10 μL micropipette (JET BIOFIL^®^, Seoul, Republic of Korea) at a flow rate of 3 μL/min. Animals were given one-minute intervals to regain normal respiratory function. All the effects were evaluated 21 days later.

### 4.5. GRP78 Labeling and Confocal Microscopy

GRP78 protein was conjugated with a fluorescent Alexa-555 dye (Invitrogen, Waltham, MA, USA) according to the manufacturer’s protocol. Briefly, 50 µL of 10 mg/mL Alexa-555 solution in dimethyl sulfoxide was slowly added to 5 mg of GRP78 in 500 µL of 0.1 M sodium bicarbonate, pH 8.3, and vortexed for 2 min. The mixture was incubated for 1 h at 4 °C with continuous stirring. The reaction was stopped by adding 50 µL of freshly prepared 1.5 M hydroxylamine, pH 8.5. The conjugate was separated from non-reacted labels through triple dialysis in PBS at 4 °C.

GRP78 protein labeled with Alexa-555 was administered intranasally to rats (*n* = 4) at a dose of 1.6 μg/8 μL, 4 h after a microinjection of LC or after PBS injection (*n* = 4) into SNpc. Animals after LC-injection into SNpc treated with unlabled-GRP78 (*n* = 4) were used as controls. Three hours later, the rats were anesthetized with Zoletil-100 (50 mg/kg, i.m.) and rapidly transcardially perfused with 0.1 M PBS (pH = 7.4) and 4% paraformaldehyde in 0.1 M PBS. After that, the animals were decapitated, and their brains were isolated and placed in the same fixative overnight at 4 °C. Following 48 h incubation in 30% sucrose/PBS at 4 °C for cryoprotection, the brains were frozen in cold isopentane (−42 °C) and stored at −80 °C for further use. Serial frontal brain sections were prepared using a Leica CM-1520 cryostat (“Leica Biosystems”, Nussloch, Germany). Sections (10 and 20 μm) of the SNpc, the ventral tegmental area (VTA), and the locus coeruleus were prepared according to the brain atlas [74]. Eight to twelve alternate series of sections were mounted on SuperFrost Plus Adhesion Microscope Slides (“Gerhard Menzel GmbH”, Braunschweig, Germany) and stored at −22 °C.

For confocal microscopy, brain sections were dried at 23 °C overnight, repeatedly washed in PBS or PBS with 0.1% Tween-20 (PBST), and pre-incubated in 4% blocking solution (2% bovine serum albumin and 2% normal goat serum diluted in PBST) for 1 h at 23 °C. Next, the sections were incubated with primary antibodies against tyrosine hydroxylase (TH; 1:900; rabbit, ab117112, Abcam, Cambridge, UK), GRP78 (1:300; rabbit, ab21685, Abcam, Cambridge, UK), or Iba (1:500; rabbit, Novus Biologicals, Centennial, CO, USA) for 24 h. After washing with PBS, the sections were incubated for 2 h at room temperature with secondary anti-rabbit IgG antibodies labeled with DyLight-488 (1:350; 35552, Thermo Scientific, Waltham, MA, USA). Following several PBS washes, the slides were coverslipped with Mowiol (Sigma, Burlington, MA, USA). Unlabeled sections were used to measure autofluorescence. Images were obtained on a DMI6000 confocal microscope with a Leica TCS SP5 laser scanning confocal setup (Leica Microsystems, Wetzlar, Germany) using a ×63 oil immersion objective. The resulting images were analyzed using the Leica LAS AF version 4.0 software package. To avoid cross-interference between fluorochromes, images for Alexa-555 and DyLight-488 were acquired using the sequential image recording method.

### 4.6. Immunohistochemical Studies

21 days after the first LC microinjection, rats were anesthetized with Zoletil-100 and decapitated. One half of each brain was used for immunohistochemical assays. The second half was used for further biochemical analysis. For immunohistochemical assays, brains were isolated and placed in the 4% paraformaldehyde in 0.1 M PBS overnight at 4 °C. Following 48 h incubation in 30% sucrose/PBS at 4 °C, the brains were frozen in cold isopentane (−42 °C) and stored at −80 °C for further use. Serial frontal brain sections were prepared using a Leica CM-1520 cryostat (“Leica Biosystems”, Nussloch, Germany). Sections (10 μm) of the SNpc were prepared according to the brain atlas [74]. Ten to twelve alternate series of sections were mounted on SuperFrost Plus Adhesion Microscope Slides (“Gerhard Menzel GmbH”, Braunschweig, Germany) and stored at −22 °C.

For bright-field microscopic analysis, brain sections were dried at 23 °C overnight. Next, sections were repeatedly washed in PBS and were preincubated first with 3% H_2_O_2_–10% methanol for 20 min, and then with 4% blocking solution for 1 h at room temperature. Sections were incubated for 48 h at room temperature with primary antibodies against microglia marker Iba-1 (1:500; rabbit, Novus Biologicals, Centennial, CO, USA) or anti-GRP78 antibodies (1:400, rabbit, Affinity Biosciences, Zhenjiang, China). Following incubation with biotinylated secondary antibodies, sections were incubated with streptavidin–peroxidase (1:250; Vector Labs., Newark, CA, USA). Reactions were visualized using 3,3-diamino-benzidine (Sigma-Aldrich, St. Louis, MO, USA) as a chromogen. To ensure specificity of the immunohistochemical staining, staining reactions lacking primary antibodies were performed.

Images of the stained sections of SNpc were obtained using a Zeiss Axio Imager A1 microscope (Carl Zeiss, Jena, Germany) with a built-in camera and Axio-Vision 4.8 software. Quantitative analysis was performed using 10–12 sections from each animal at the same level of the studied zones, separated by approximately 70 μm. The number of cells accounted for a standard area of tissue captured by a light microscope camera using ×20 lens—697 × 523 µm for Iba staining, and ×10 lens—1389 × 1040 µm for GRP78 staining. The number of Iba-positive cell bodies was counted manually and expressed as the average number of positive stained microglia cells per SNpc section. The optical density reflecting the content of an GRP78-immunopositive substance was calculated as the difference between intensely colored neurons containing an immunoreactive substance and the intensity of background coloring (not containing an immunoreactive substance) on the same section. The results were presented in relative units of optical density.

The analysis was performed using the PhotoM freeware version 1.21 (http://www.t_lambda.chat.ru/ accessed on 11 December 2019).

### 4.7. Immunoblotting

The SNpc was dissected from the brain according to the brain atlas [74]. All samples were weighed, frozen at −80 °C, and stored until the analysis. SNpc tissue was then homogenized in lysis buffer containing 20 mM Tris–HCl (pH 7.5), 150 mM NaCl, 0.5% Triton X-100, and 2 mM EDTA supplemented with a protease inhibitor cocktail (Sigma Aldrich, St. Louis, MO, USA) and a phosphatase inhibitor cocktail (Roche, Basel, Switzerland). Next, homogenized tissue was incubated on ice for 1 h until the lysis of the samples was complete. Following centrifugation (13,500× *g* for 10 min), the supernatant was used for protein quantification and further assays. Protein concentration was measured by the Lowry assay with BSA as a standard. For Western blotting, the protein supernatant was mixed 2:1 with loading buffer (0.0625 M Tris–HCl (pH 6.8), 10% glycerol, 2% SDS, 0.1 mM EDTA, 0.006% bromophenol blue, 10% β-mercaptoethanol) and heated at 95 °C for 7 min. Equal volume aliquots containing 30 μg of total protein were loaded onto 11% polyacrylamide gel and separated by electrophoresis with the Precisions Plus Protein Dual Xtra Standards marker (BioRad, Hercules, CA, USA). Protein bands were then transferred onto PVDF membranes (pore size 0.2 μm; BioRad, Hercules, CA, USA) by wet transfer with a TransBlot device (BioRad, Hercules, CA, USA).

To prevent nonspecific antibody binding, membranes were incubated in a blocking solution (PBS added with 0.1% Tween-20 and 3% BSA or PBS added with 0.1% Tween-20 and 5% Nonfat dairy milk, NFDM) for 1 h. The membranes were then incubated at 4 °C overnight in PBST solution containing 0.05% NaN_3_ and 3% BSA or 5% NFDM and one of the following primary antibodies: monoclonal antibodies against GRP78 (1:1000, rabbit, ab21685, Abcam, Cambridge, UK), phospho-NFkB p65 (Ser536) (1:750, rabbit, mAb#3033, Cell Signaling, Danvers, MA, USA), NFkB p65 (1:750, rabbit, mAb#8242, Cell Signaling, Danvers, MA, USA), or polyclonal antibodies against phospho-eIF2α (Ser51) (1:750, rabbit, AF3087, Affinity Biosciences, Zhenjiang, China), eIF2α (1:1000, rabbit, ab5369 Abcam, Cambridge, UK), CHOP (1:1000, rabbit, DF6025 Affinity Biosciences, Zhenjiang, China), caspase-9 cleaved (Asp353) (1:1000, rabbit, AF6348, Affinity Biosciences, Zhenjiang, China), caspase-3 cleaved (Asp175) (1:1000, rabbit, AF7022 Affinity Biosciences, Zhenjiang, China), phospho-α-syn (Ser129) (1:1500, rabbit, AF3285, Affinity Biosciences, Zhenjiang, China), α-syn (1:1200, rabbit, AF6285, Affinity Biosciences, Zhenjiang, China), Interleukine-6 (1:1000, rabbit, MAA079Ra21, Affinity Biosciences, Zhenjiang, China), or TNF-α (1:1000, rabbit, Affinity Biosciences, Zhenjiang, China). The polyclonal antibodies against GAPDH (1:1000, rabbit, AF7021 Affinity Biosciences, Zhenjiang, China) and β-Actin (1:1000, mouse, sc-69879, Santa Cruz Biotechnology Inc, Dallas, TX, USA) were used to visualize loading controls. Then, membranes were washed with PBST three times for 10 min each and incubated at room temperature for 1 h with a PBS solution of secondary antibodies conjugated with horseradish peroxidase (1:10,000, HRP-Goat Anti-Rabbit IgG, 111-035-144, or HRP-Donkey Anti-Mouse IgG, 715-036-150, both Jackson ImmunoResearch Europe Ltd., Ely, UK). After three washes with PBST, bands were visualized using a chemiluminescence protocol with the Novex ECL HRP Chemiluminescent Substrate Reagent Kit (Invitrogen, Carlsbad, CA, USA) and a ChemiDoc MP imager (Bio-Rad, Hercules, CA, USA).

Protein levels were normalized to the GAPDH or β-Actin signal. The relative amounts of phospho-eIF2α (Ser51) or phospho-NFkB p65 (Ser536) were determined by adjusting for total eIF2α or NFkB p65 protein or for GAPDH. Densitometric analysis was performed in the open-source ImageJ 1.8 software (National Institutes of Health, New York, NY, USA). The ratios of the optical densities of specific protein bands to the total protein were compared to the mean of the control group.

### 4.8. Statistics

All data were analyzed using GraphPad Prism 8 (GraphPad Software, San Diego, CA, USA). The distribution normality was checked using the Kolmogorov–Smirnov test. Multiple comparisons between the groups of rats were run using the two-way ANOVA test followed by Tukey’s post hoc tests. Intergroup differences were considered statistically significant at *p* ≤ 0.05. All data were represented as the mean ± standard error of the mean (SEM) and as individual values.

## 5. Conclusions

In an LC-induced rodent model, we achieved the first successful treatment of PD-like pathology using an intranasal delivery of the recombinant human protein GRP78 to the brain. We report that intranasally administered GRP78 rapidly enters affected brain regions and prevents the development of neurodegeneration in the nigrostriatal system during the preclinical stage of PD in rats. LC-induced disturbances, including ER stress-dependent apoptosis and the abnormal accumulation of monomeric phosphorylated pS129-α-syn, are alleviated with GRP78 administration. Moreover, exogenous GRP78 exhibits anti-inflammatory properties and can protect neurons against the excessive activation of microglia, as well as the increased production of pro-inflammatory cytokines, TNF-α and IL-6, by targeting NF-κB signaling pathways. Although further investigation into molecular mechanisms is still necessary, we anticipate that this therapeutic approach, based on intranasal exogenous GRP78, may contribute to the development of effective therapeutic agents for PD and other synucleinopathies in the future.

## Data Availability

The data presented in this study are available on request from the corresponding author M.B.P. and I.V.E.

## Supplementary Materials

The following supporting information can be downloaded at: https://www.mdpi.com/article/10.3390/ijms25073951/s1.

## Abbreviations

ATF6	activating transcription factor 6
α-syn	α-synuclein
CHOP	C/EBP homologous protein
DA	dopaminergic
eIF2α	eukaryotic initiation factor 2α
ERAD	ER-associated degradation
ER	endoplasmic reticulum
GAPDH	glyceraldehyde 3-phosphate dehydrogenase
GRP78/BiP	glucose-regulated protein 78/immunoglobulin heavy chain binding protein
HSP	heat-shock protein
IRE1α	inositol-requiring transmembrane kinase/endoribonuclease 1α
IL-6	Interleukin-6
Iba1	Ionized calcium-binding adaptor molecule 1
LC	lactacystin
MPTP	1-methyl-4-phenyl-1,2,3,6-tetrahydropyridine
NF-κB	nuclear factor kappa-light-chain-enhancer of activated B cells
PD	Parkinson’s disease
