# Supplementary material for: Intranasal Administration of GRP78 Protein (HSPA5) Confers Neuroprotection in a Lactacystin-Induced Rat Model of Parkinson’s Disease
Source: Int J Mol Sci. 2024 Apr 2;25(7):3951. doi: 10.3390/ijms25073951 (PMC11011682; doi:10.3390/ijms25073951)

IMAGES WITH COMMENTS FOR FIGURE 3:

Western blot of the nigral samples of rats from experimental groups: Vehicle group; Grp78 treatment alone; lactacystin model of PD; Grp78 treatment in lactacystin-induced model of PD.

Full blots stained by Ponceau solution:

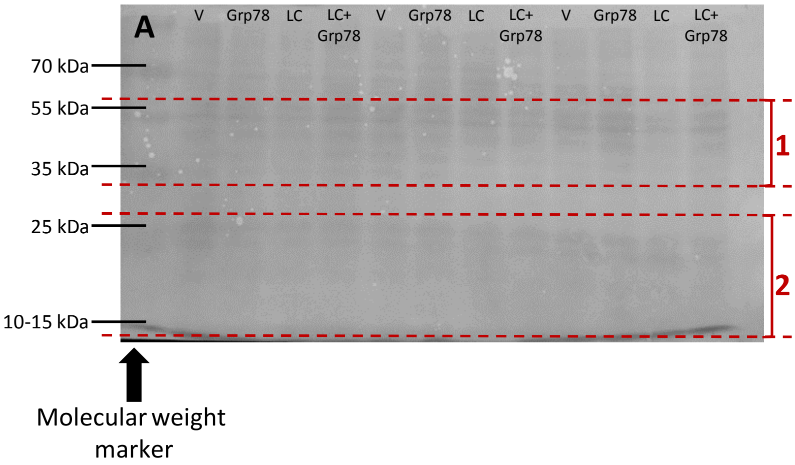

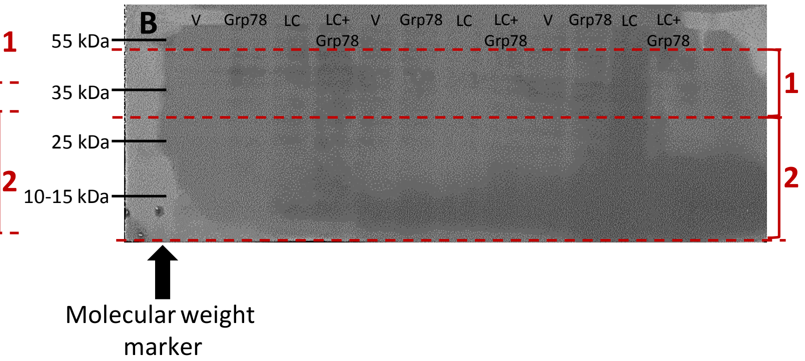

V- Vehicle group; Grp78 – Grp78 alone; LC – lactacystin model of PD; LC+Grp78 – Grp78 in lactacystin-induced model of PD.

Sample order: V, Grp78, LC, LC+Grp78, V, Grp78, LC, LC+Grp78, V, Grp78, LC, LC+Grp78.

After blotting, the membrane was cut into two pieces according to the molecular weights of proteins of interest.

1. Piece #1: GAPDH protein was detected.

- Original image of GAPDH; bands used for representative blot are highlighted with black frame.

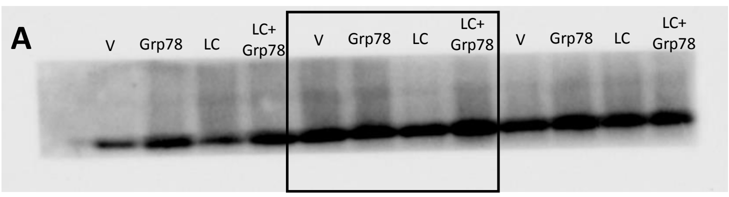

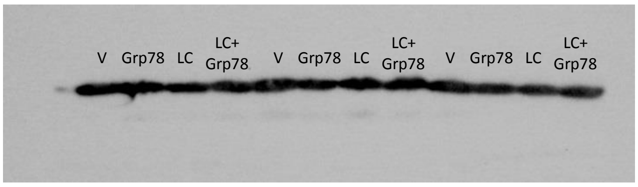

2. Piece #2: soluble form of α-syn and α-syn phosphorylated at S129 (pS129) were detected.

Detection order: α-syn phosphorylated at S129 (pS129) → mild stripping procedure → soluble α-syn .

- Original image of pS129-α-syn ; bands used for representative blot are highlighted with black frame.

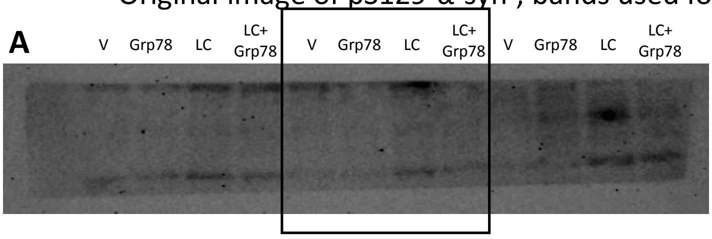

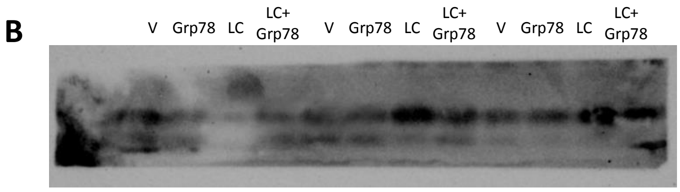

- Original image of soluble-α-syn; bands used for representative blot are highlighted with black frame.

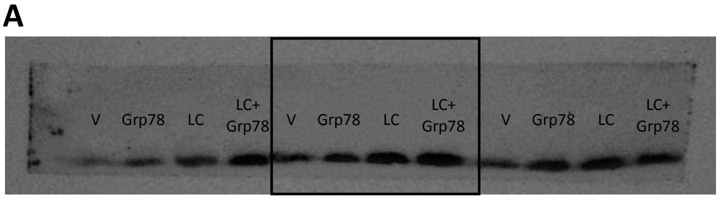

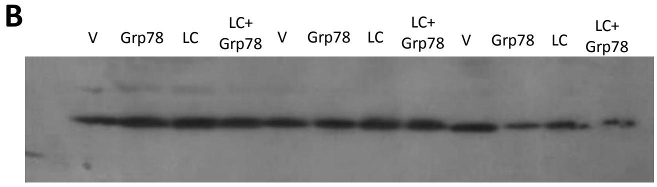

IMAGES WITH COMMENTS FOR FIGURE 4:

Western blot of the nigral samples of rats from experimental groups: Vehicle group; Grp78 treatment alone; lactacystin model of PD; Grp78 treatment in lactacystin-induced model of PD.

Full blot stained by Ponceau solution:

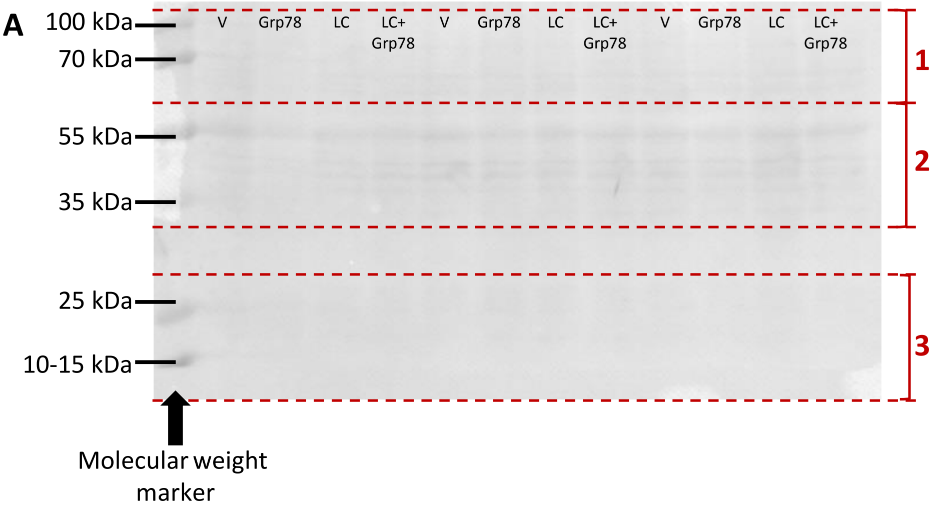

V- Vehicle group; Grp78 – Grp78 alone; LC – lactacystin model of PD; LC+Grp78 – Grp78 in lactacystin-induced model of PD.

Sample order: V, Grp78, LC, LC+Grp78, V, Grp78, LC, LC+Grp78, V, Grp78, LC, LC+Grp78.

After blotting, the membrane was cut into three pieces according to the molecular weights of proteins of interest.

1. Piece #1: GRP78 protein was detected.

- Original image of GRP78; bands used for representative blot are highlighted with black frame.

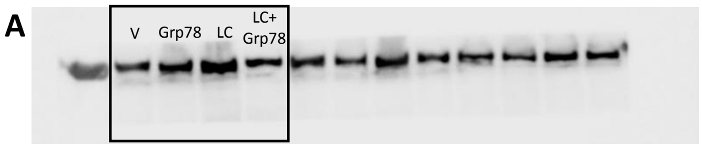

2. Piece #2: eF2α; pSer51-eIF2a; cleaved caspase-9 and β-Actin were detected

Detection order: cleaved caspase-9 → mild stripping procedure → pSer51-eIF2α → mild stripping procedure → β-Actin → mild stripping procedure → eF2α

- Original image of cleaved caspase-9; bands used for representative blot are highlighted with black frame.

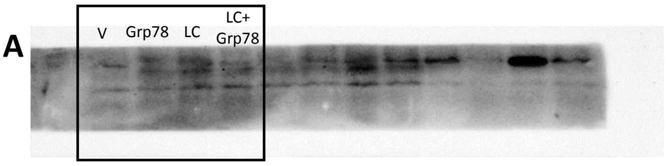

- Original image of pSer51-eIF2α; bands used for representative blot are highlighted with black frame.

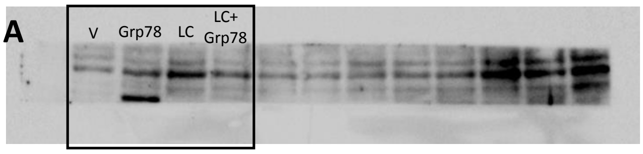

- Original image of β-Actin ; bands used for representative blot are highlighted with black frame.

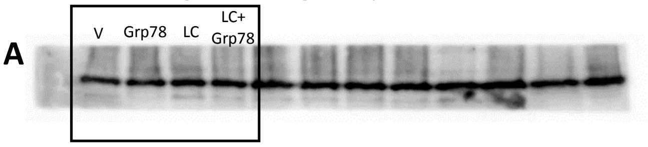

- Original image of eIF2α ; bands used for representative blot are highlighted with black frame.

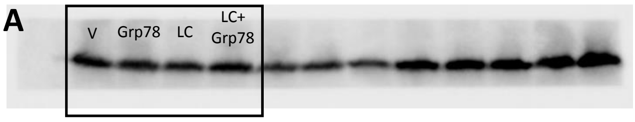

3. Piece#3: cleaved caspase-3 and CHOP were detected

Detection order: cleaved caspase-3 → mild stripping procedure → CHOP

- Original image of cleaved caspase-3; bands used for representative blot are highlighted with black frame.

A

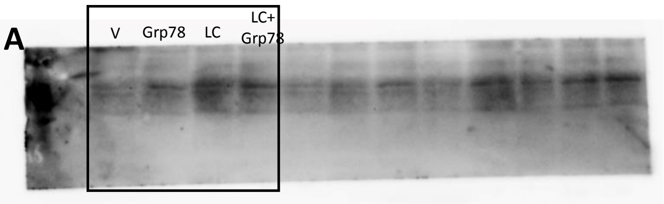

- Original image of CHOP; bands used for representative blot are highlighted with black frame.

A

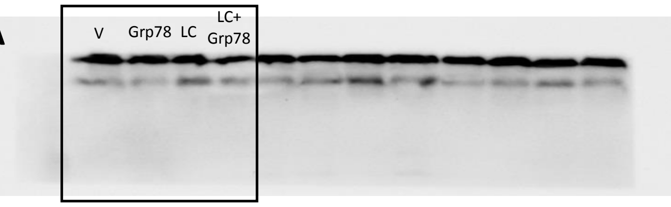

IMAGES WITH COMMENTS FOR FIGURE 6:

Western blot of the nigral samples of rats from experimental groups: Vehicle group; Grp78 treatment alone; lactacystin model of PD; Grp78 treatment in lactacystin-induced model of PD.

Full blot stained by Ponceau solution:

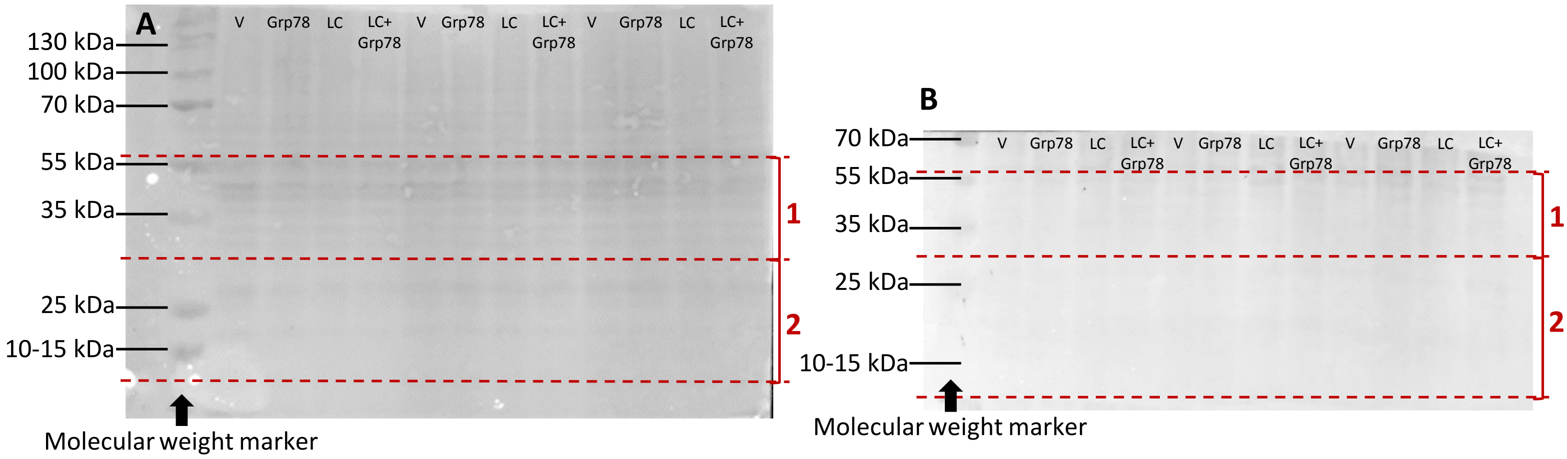

V- Vehicle group; Grp78 – Grp78 alone; LC – lactacystin model of PD; LC+Grp78 – Grp78 in lactacystin-induced model of PD.

Sample order: V, Grp78, LC, LC+Grp78, V, Grp78, LC, LC+Grp78, V, Grp78, LC, LC+Grp78.

After blotting, the membrane was cut into three pieces according to the molecular weights of proteins of interest.

1. Piece #1: GAPDH was detected

- Original image of GAPDH; bands used for representative blot are highlighted with black frame.

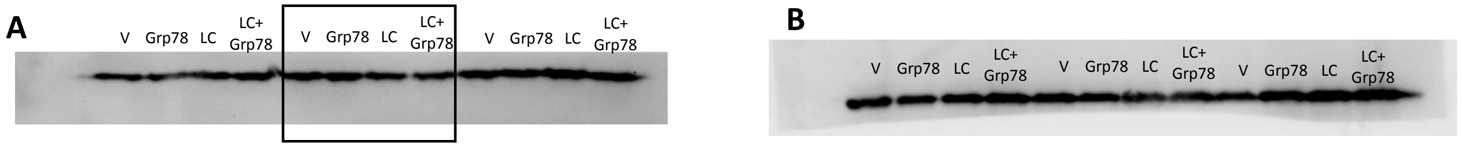

2. Piece#2: TNF and IL-6 were detected

Detection order: TNFα→ mild stripping procedure → IL-6

- Original image of TNFα; bands used for representative blot are highlighted with black frame.

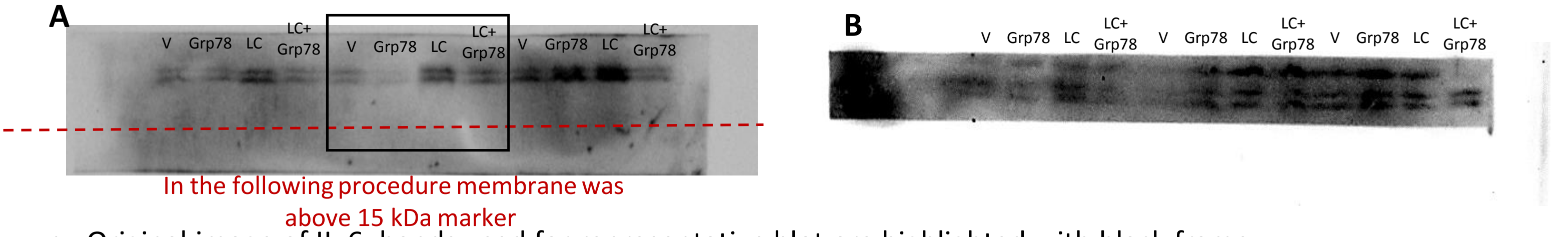

- Original image of IL-6; bands used for representative blot are highlighted with black frame.

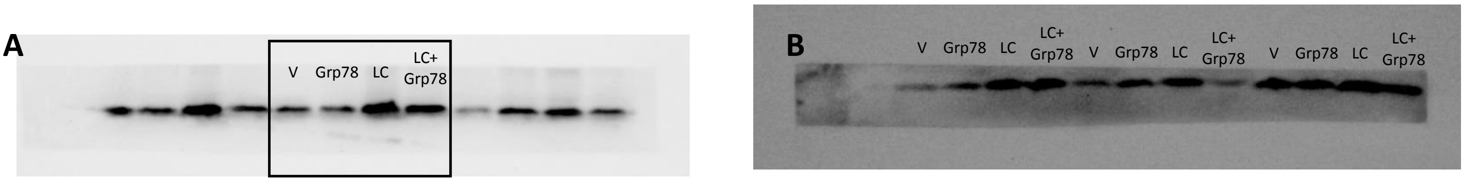

IMAGES WITH COMMENTS FOR FIGURE 7:

Western blot of the nigral samples of rats from experimental groups: Vehicle group; Grp78 treatment alone; lactacystin model of PD; Grp78 treatment in lactacystin-induced model of PD.

Full blot stained by Ponceau solution:

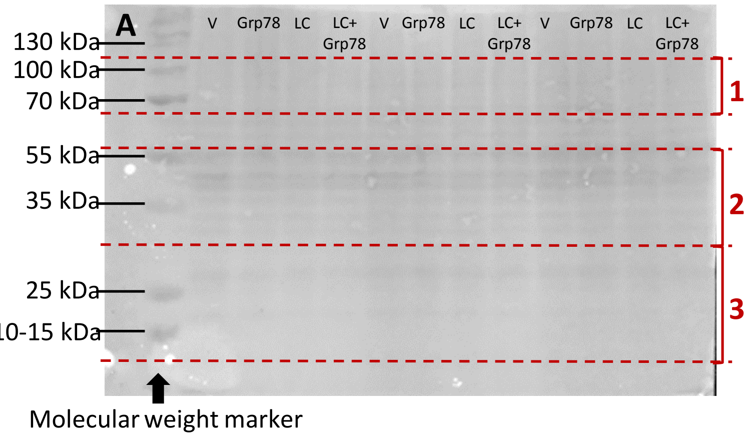

V- Vehicle group; Grp78 – Grp78 alone; LC – lactacystin model of PD; LC+Grp78 – Grp78 in lactacystin-induced model of PD.

Sample order: V, Grp78, LC, LC+Grp78, V, Grp78, LC, LC+Grp78, V, Grp78, LC, LC+Grp78.

After blotting, the membrane was cut into three pieces according to the molecular weights of proteins of interest.

1. Piece #1: NF-kB p65 and phosphorylated NF-kB p65 (Ser536) were detected

Detection order: phosphorylated NF-kB p65 (Ser536) → mild stripping procedure → NF-kB p65

- Original image of NF-kB p65 (Ser536); bands used for representative blot are highlighted with black frame.

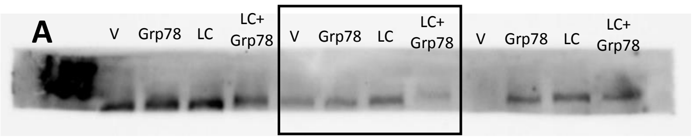

- Original image of NF-kB; bands used for representative blot are highlighted with black frame.

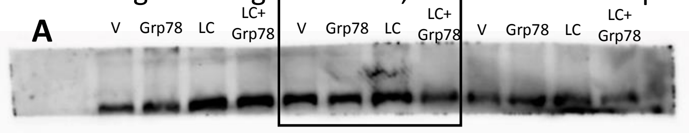

2. Piece #2: GAPDH was detected

- Original image of GAPDH; bands used for representative blot are highlighted with black frame.

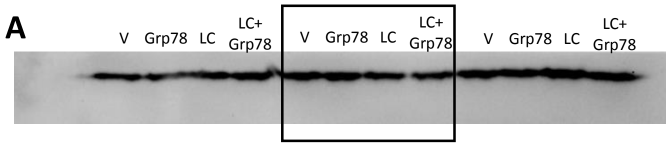

Supplement: Supplementary file 1 [file ijms-25-03951-s001.zip › Original blots.pdf]
