# Supplementary material for: Intranasal Administration of GRP78 Protein (HSPA5) Confers Neuroprotection in a Lactacystin-Induced Rat Model of Parkinson’s Disease
Source: Int J Mol Sci. 2024 Apr 2;25(7):3951. doi: 10.3390/ijms25073951 (PMC11011682; doi:10.3390/ijms25073951)
Supplement: Supplementary file 1 [file ijms-25-03951-s001.zip › Supplement.pdf]

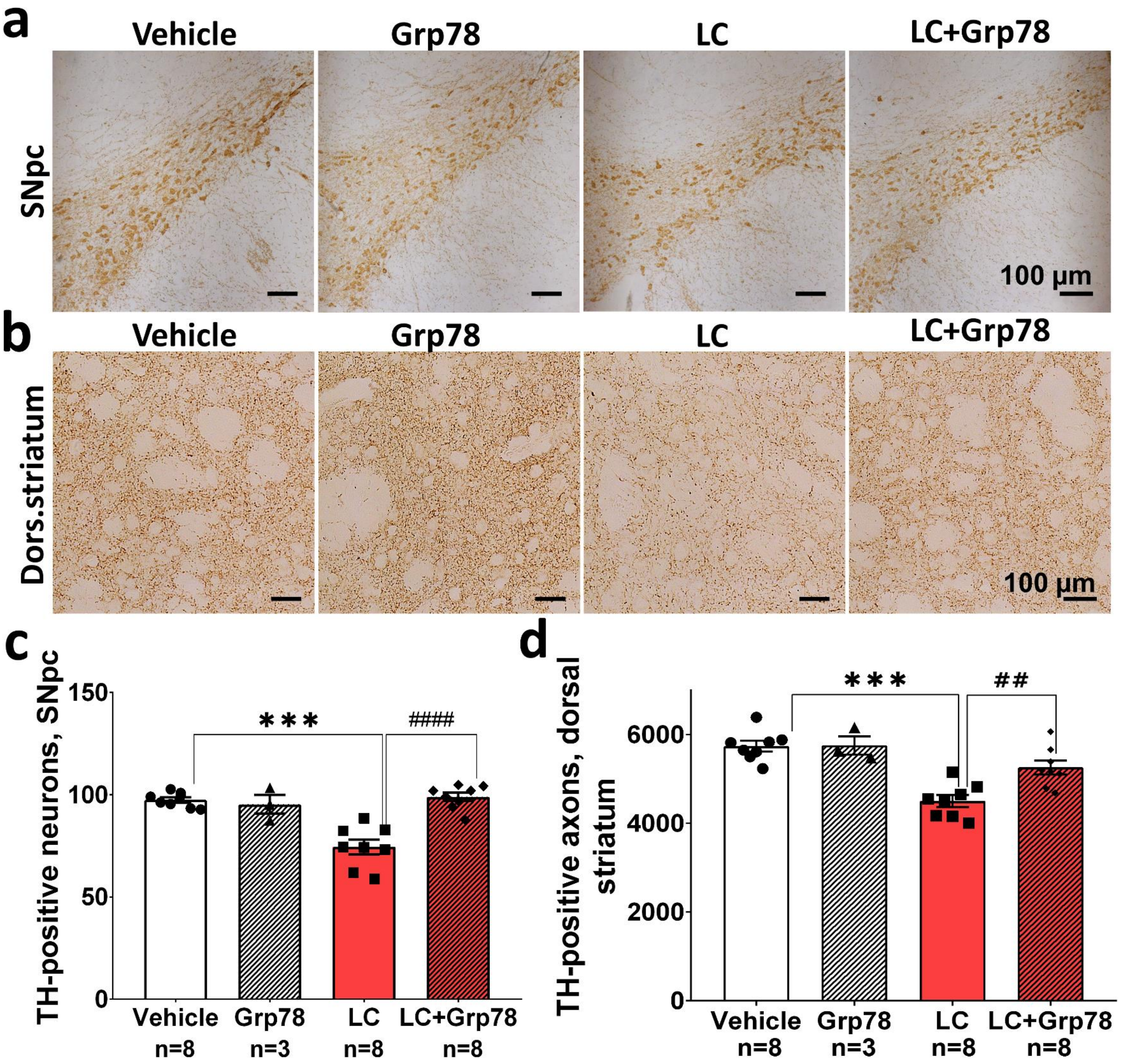

Figure S1. Treatment of rats in the preclinical stage of PD-like neurodegeneration with exogenous GRP78 prevents the development of the neurodegenerative process in the nigrostriatal system. (**a**) Sections (10 µm) of the substantia nigra pars compacta (SNpc) and (**b**) the dorsal stri-atum (Dors. striatum) from experimental groups were prepared according to the brain atlas and stained with antibodies against tyrosine hydroxylase (1:900; rabbit, Abcam, UK). The images were obtained using a Zeiss Axio Imager A1 microscope (Carl Zeiss, Germany) with a built-in camera and Axio-Vision 4.8 software. Scale bars - 100 µm as indicated in the figure. (**c, d**) Quantitative analysis was performed on 10–12 sections from each animal at the same level of the studied zones, separated by approximately 70 µm. The analysis was performed using the PhotoM freeware (http://www.t_lambda.chat.ru/). Eight animals from the Control, LC model, and LC model with GRP78 treatment groups, as well as three animals treated only with GRP78, were used for analysis. The columns indicate mean values with standard errors. The dots present individual values per rat. Two-way ANOVA followed by Tukey's post hoc analysis was per-formed to determine the effects of GRP78 therapy. Interaction factor for SNpc $F (1, 23) = 19.50$ $p = 0.0002$, Grp78 factor for SNpc $F (1, 23) = 13.62$ $p = 0.0012$; LC factor for SNpc $F (1, 23) = 10.31$ $p = 0.0039$. Interaction factor for Dorsal striatum $F (1, 23) = 5.093$ $p = 0.0338$, Grp78 factor for Dorsal striatum $F (1, 23) = 5.541$ $p = 0.0275$; LC factor for Dorsal striatum $F(1, 23) = 28.04$ $p < 0.0001$. Asterisks indicate significant differences between groups according to Tukey's post hoc tests: *** $p < 0.001$ vs. the vehicle group; ## $p < 0.01$, #### $p < 0.0001$ vs. the LC group.

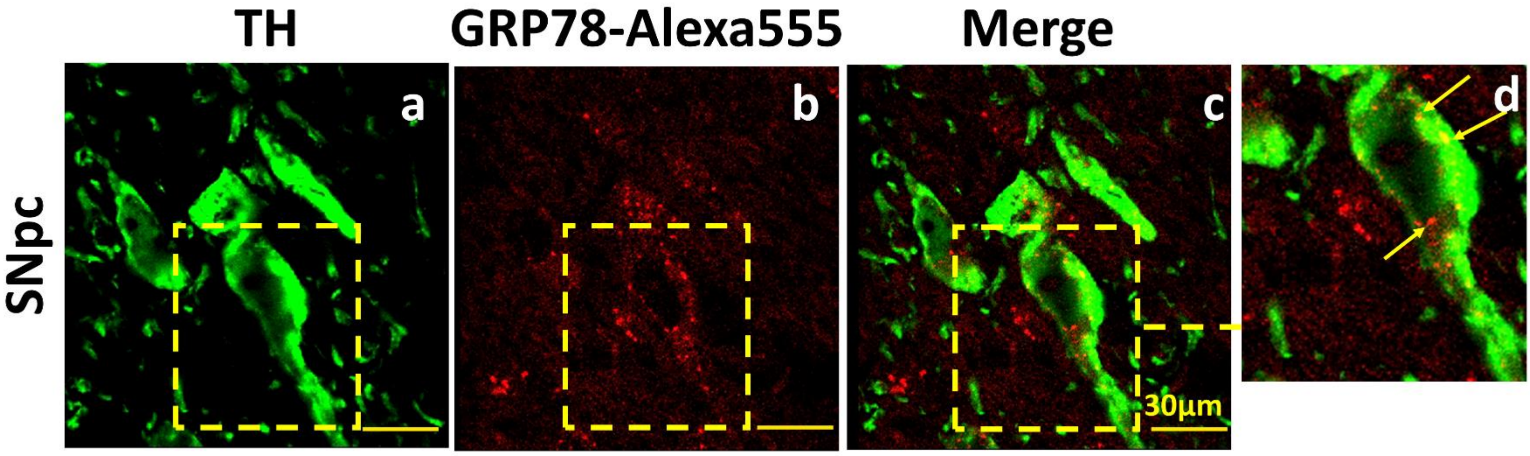

Figure S2. Labeled GRP78 penetrates into the substantia nigra pars compacta (SNpc) and is localized in dopaminergic neurons of the 3 h after its intranasal administration in a control rat. Brain sections of SNpc were stained with specific anti-TH antibodies (a, green signal). (b) Localization of labeled GRP78 is seen as a red signal. (c) Panel shows co-localization of labeled GRP78 and anti-TH signals. (d) Panel shows magnified representative images of the co-localization. Images were obtained using confocal microscopy. Scale bars are 30 μm.

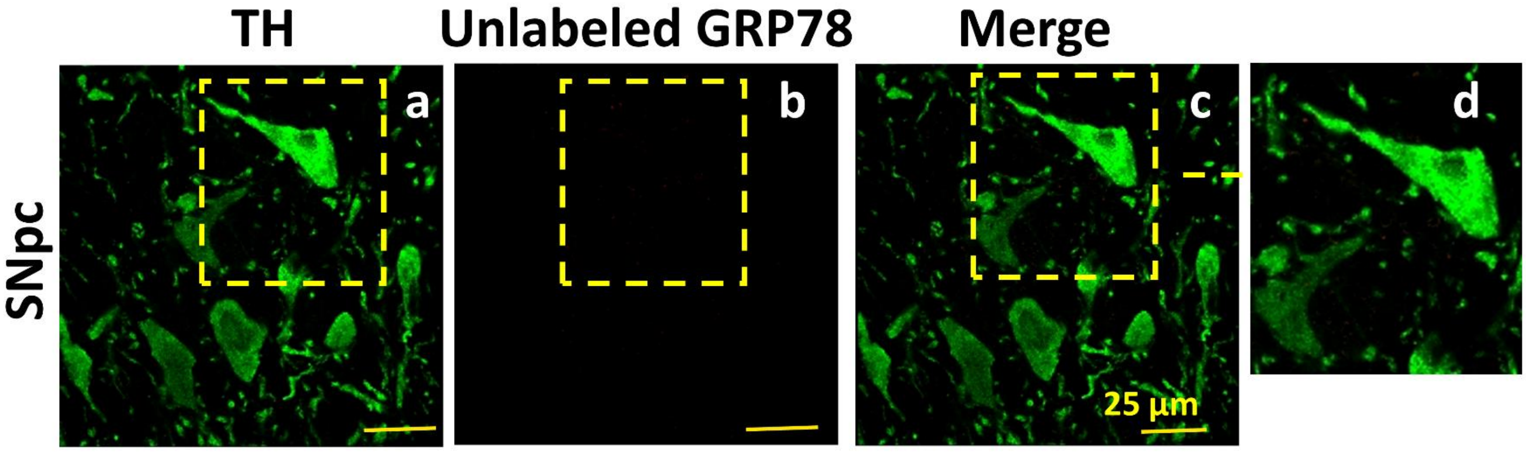

Figure S3. Red fluorescent signal was absent following the intranasal administration of unlabeled GRP78. Brain sections of SNpc were stained with specific anti-TH antibodies (**a,** green signal). (**b**) Absence of red signal (**c**) Panel shows absence of colocalized signal. (**d**) Panel shows magnified representative image. Images were obtained using confocal microscopy. Scale bars are 25 μm.

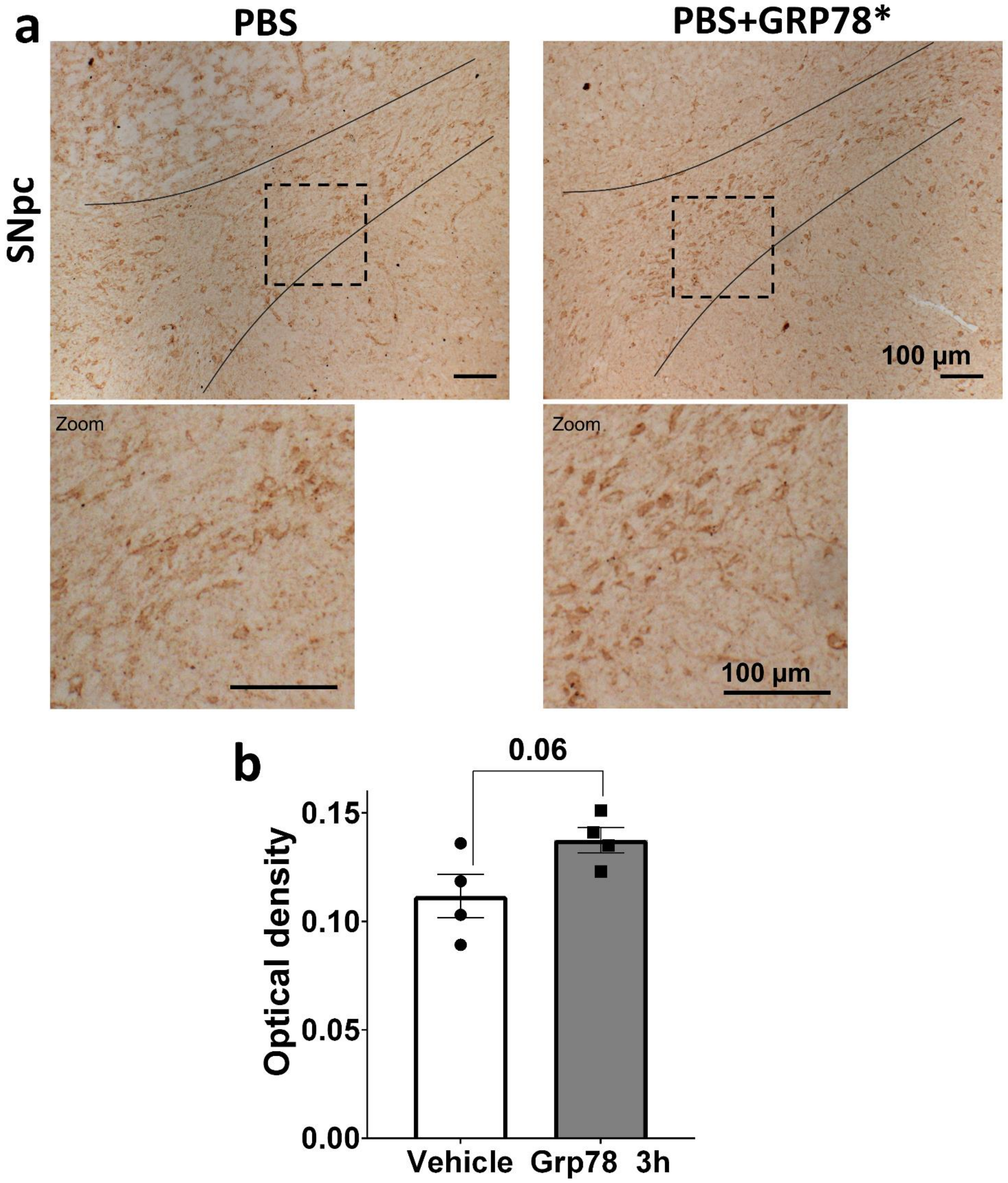

Figure S4. Exogenous GRP78 accumulates in live brain neurons of the SNpc during the first 3 h after intranasal administration in control rats. (**a**) Representative images of animals treated with solvent (PBS) and healthy controls treated labeled GRP78 (PBS+GRP78*). Lower panels show magnified images (Zoom). Brain sections were stained with anti-GRP78 antibodies (1:400, rabbit, Affinity Biosciences, China). The images were obtained using a Zeiss Axio Imager A1 microscope (Carl Zeiss, Germany) with a built-in camera and Axio-Vision 4.8 software. Scale bars - 100 μm as indicated in the figure. **(b**) Quantitative analysis was performed on 10–12 sections from each animal at the same level of the studied zones, separated by approximately 70 μm. The analysis was performed using the PhotoM freeware (http://www.t_lambda.chat.ru/). The optical density reflecting the content of an GRP78-immunopositive substance was calculated as the difference between intensely colored neurons containing an immuno-reactive substance and the intensity of background coloring (not containing an immunoreactive substance) on the same section. The results were presented in relative units of optical density. Four animals from the PBS and PBS+GRP78 were used for analysis. The columns indicate mean values with standard errors. The dots present individual values per rat. T-test analysis was performed to determine the effects of GRP78 administration.

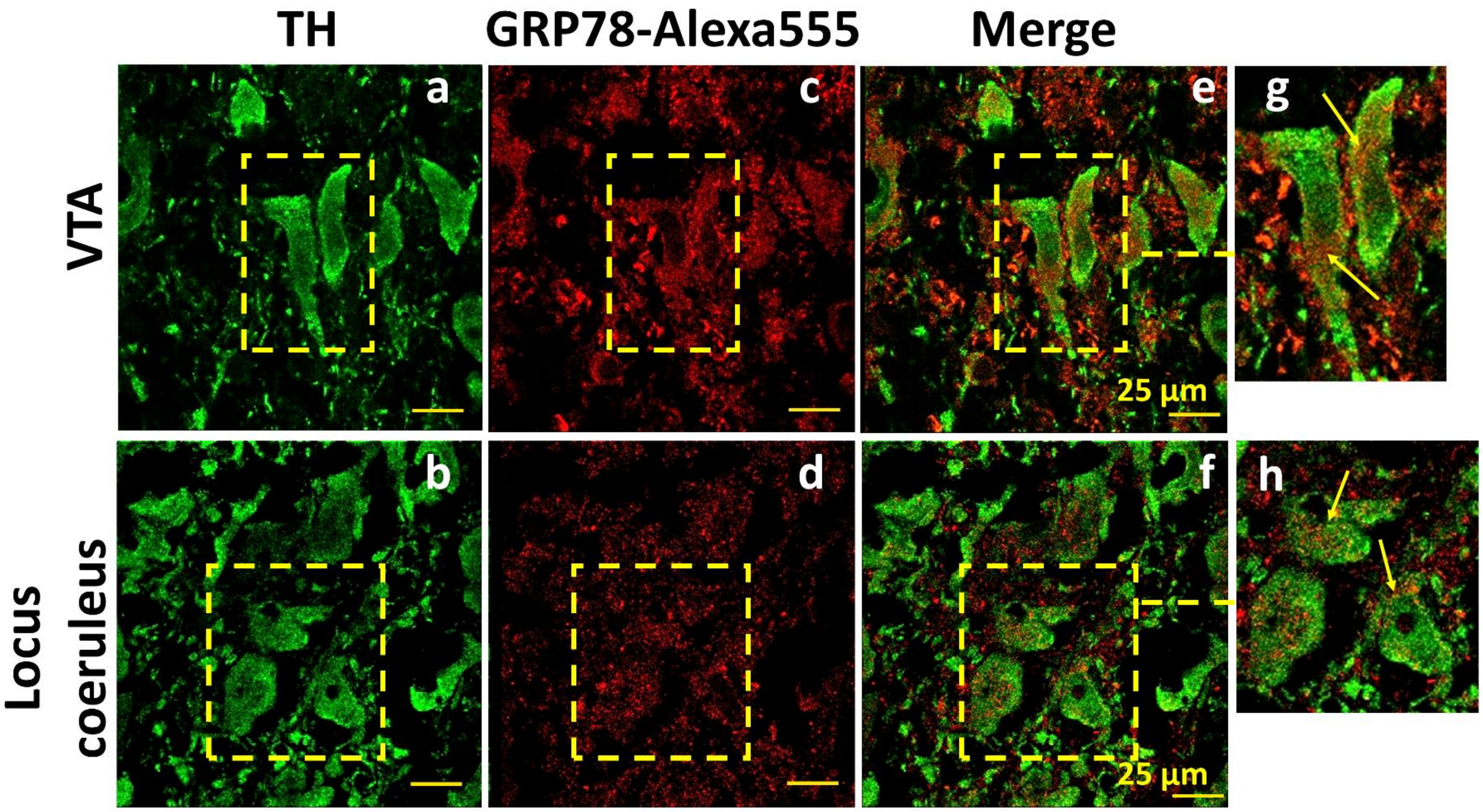

Figure S5. Labeled GRP78 migrated to ventral tegmental area (VTA) and locus coeruleus 3 h after its intranasal administration in a rat model of Parkinson's disease. Brain sections of (**a**) VTA and (**b**) locus coeruleus were stained with specific anti-TH antibodies. (**c, d**) Localization of la-beled GRP78 is seen as a red signal. (**e, f**) Panels show co-localization of labeled GRP78 and an-ti-TH signals. (g, h) Panels show magnified representative images of the co-localization. Images were obtained using confocal microscopy. Scale bars are 25 μm.
